# Community Care Needs of Highly Complex Chronic Patients: An Epidemiological Study in a Healthcare Area

**DOI:** 10.3390/nursrep14020096

**Published:** 2024-05-20

**Authors:** Pedro Ruymán Brito-Brito, Martín Rodríguez-Álvaro, Domingo Ángel Fernández-Gutiérrez, Janet Núñez-Marrero, Antonio Cabeza-Mora, Alfonso Miguel García-Hernández

**Affiliations:** 1 Nursing Department, Faculty of Healthcare Sciences, University of La Laguna, 38200 Santa Cruz de Tenerife, Spain; pbritobr@ull.edu.es (P.R.B.-B.); almigar@ull.edu.es (A.M.G.-H.); 2 Health Services Management Board of La Palma, The Canary Islands Health Service, 38713 Breña Alta, Spain; 3 Primary Care Management Board of Tenerife, The Canary Islands Health Service, 38003 Santa Cruz de Tenerife, Spain; jnunmar@gobiernodecanarias.org; 4 Primary Care Management Board of Gran Canaria, The Canary Islands Health Service, 35006 Las Palmas de Gran Canaria, Spain; acabmorc@gobiernodecanarias.org

**Keywords:** primary health care, standardised nursing terminology, epidemiology, needs assessment, health information systems, community health nursing

## Abstract

One of the priorities in family and community care is the epidemiological surveillance of the care needs and dysfunctionality present in populations of highly complex chronic patients (HCCPs) using standardised nursing languages. The aim of this study is to establish the prevalence of care needs and dysfunctionality among HCCPs in a specific health area by municipalities and geographical areas (metropolitan, north, and south) while verifying correlations with sociodemographic, financial, and health characteristics. This is an epidemiological, observational, descriptive, cross-sectional study carried out with a sample of 51,374 HCCPs, whose data were grouped into 31 municipalities. Data were collected on the following variables: sociodemographic, financial, health, functional status (health patterns), and care needs (nursing diagnoses). The mean age of the HCCPs was 73.41 (1.45) years, of which 56.18 (2.86)% were women. The municipalities in the northern area have a significantly higher proportion of older patients, HCCPs, lower incomes, and higher unemployment rates. The southern area had higher proportions of non-Spanish nationals and professionals in the hotel and catering industry, and the metropolitan area had a higher proportion of employed individuals and higher levels of education. Northern municipalities had a higher prevalence of illnesses and anxiolytic and anti-psychotic treatments. Dysfunctionality frequencies did not differ significantly by area. However, a higher prevalence of 13 nursing diagnoses was observed in the north. A high number of correlations were observed between population characteristics, dysfunctionality, and prevalent diagnoses. Finally, the frequencies of dysfunctionality in the population and the most common care needs were mapped by municipality. This research sought to ascertain whether there was an unequal distribution of these two aspects among HCCPs in order to gain a deeper epidemiological understanding of them from a family and community perspective using standardised nursing languages. This study was not registered.

## 1. Introduction

Community health nursing is a synthesis of nursing practice and public health practice applied to promoting and preserving the health of populations. The practice is general and comprehensive. The dominant responsibility is to the population as a whole; nursing directed to individuals, families, or groups contributes to the health of the total population. The nurse’s actions acknowledge the need for comprehensive health planning, recognize the influences of social and ecological issues, give attention to populations at risk and utilize dynamic forces that influence change [1]. This definition was provided by the American Nurses’ Association (ANA) in 1973, an approach that is still valid half a century later.

In Spain, the curriculum for the family and community health nursing specialty [2] includes the following priority skills: (1) being able to identify the healthcare needs of the population and address them properly in any setting; and (2) being able to establish and operate sentinel surveillance networks on the epidemiology of care needs. The curriculum also suggests that the care needs of populations with prevalent chronic diseases, disabilities, risks of disease, and frailty must be adequately and effectively addressed. Primary care nurses therefore take a leading role in the community in addressing chronicity and the care needs arising from it. The Chronic Care Model (CCM) is one of the best-known models worldwide for identifying the care needs of people with chronic conditions, as it provides a suitable framework for organising healthcare services in a way that leads to satisfactory outcomes for patients and their families [3]. Following the CCM, other initiatives have been proposed by the World Health Organisation (WHO) and several authors [4,5] that take health determinants into consideration and help to coordinate interventions between the different levels of care.

In view of the impact of the COVID-19 pandemic on nursing homes and other residential care facilities, it is a priority to return to comprehensive approaches to healthcare based on models of care for the elderly and the frail, such as the Canadian Programme of Research to Integrate Services for Maintenance of Autonomy (PRISMA) [6], which seeks to preserve functional independence among frail elderly people. The Kaiser Permanente pyramid [7] is another organisational model for healthcare that is based on the stratification of the population into four large groups, as well as on service provision according to the needs identified in each of them: general population; chronic patients (70–80% of the population); high-risk patients (15%); and highly complex patients (5%). The Estrategia para el Abordaje de la Cronicidad [Strategy for Tackling Chronicity] (EAC) of the Spanish National Health System (SNHS) focuses on the latter [8] and highlights the need to strengthen primary care teams to reorganise healthcare and effectively engage patients, families, and communities in looking after their own health. Population stratification must be combined with a comprehensive assessment of patients’ medical, care, functional, and social needs.

The care needs and functional problems identified by primary care nurses in Spain are documented in the electronic health record (EHR) in accordance with a specific regulation [9], which seeks to homogenise EHRs by using the following standardised nursing languages (SNLs): NANDA-I (nursing diagnoses classification) [10], NOC (nursing outcomes classification) [11], and NIC (nursing interventions classification) [12]. Also common among nurses is the use of Gordon’s functional health patterns (HPs) [13] to assess patient functionality.

In a number of Spanish populations, such as those living in the Canary Islands, the care needs of highly complex chronic patients (HCCPs) and their levels of biopsychosocial dysfunctionality have been identified and described [14,15]. The results of these studies suggest the need for further research adopting this population-based approach, which takes geographical distribution into consideration. In the field of primary care, taking an epidemiological, community-based approach towards the health diagnosis of each population is key to gaining a better understanding of their health needs and to identifying the relationships between these needs and aspects such as the geographical context, the historical background of the community, environmental factors, demographics and population structure, living standards, social organisation, habits and attitudes, etc. [16].

NANDA-I nursing diagnoses (NDs) are constructed on the basis of a multi-axial system. Each of the seven axes represents a dimension of the human response considered in the diagnostic process. The second axis is the ‘subject of diagnosis’ and refers to the identification of problems not only at the individual level but also at the family and community levels. Similarly, the HPs framework is designed to assess individuals, families, and communities alike. In light of this and the power of geographic information systems to identify community care needs and make decisions about public health care planning [17], it is safe to say that it is worthwhile to investigate, from an epidemiological perspective, the levels of population dysfunctionality and the resulting care needs using SNLs.

The hypothesis explored in this study is that, in a healthcare area, the population distribution of dysfunctionality levels (based on HPs) and care needs (identified using NDs) is uneven in each municipality and is related to the sociodemographic, financial, and health characteristics of each community.

The objectives of this study are as follows: (1) to ascertain whether there are socio-demographic, financial, and health-related differences between municipalities depending on their corresponding healthcare area (metropolitan, north, or south); (2) to compare differences in dysfunctionality prevalence rates (by HP) and community care needs (by ND); (3) to explore whether dysfunctionality prevalence rates and community NDs are correlated with sociodemographic, financial, and health-related characteristics; and (4) to geographically represent the community distribution (by municipality) of dysfunctionality levels among HCCPs and that of care needs (based on the NDs identified as most prevalent).

## 2. Materials and Methods

### 2.1. Design and Sampling Method

This is an epidemiological study using an observational, descriptive, cross-sectional design and correlation analysis. This research used the data registered—as of 30 September 2020—in the EHRs of the entire population of HCCPs in the Tenerife Healthcare Area (Santa Cruz de Tenerife, Canary Islands, Spain), amounting to a total of 51,374 patients. These data are collected by population/community, as per the distribution of each of the 31 municipalities on the island of Tenerife.

### 2.2. Study Setting

The Tenerife Healthcare Area is one of seven healthcare areas in the Canary Islands Health Service (SCS), which is part of Spain’s National Health System and delivers public healthcare services to the population of the Canary Islands. In 2020, the total population of this autonomous community was approximately 2,173,000, with around 929,000 people living on the island of Tenerife. There are a total of 103 primary care facilities, staffed by 795 nurses and 797 family physicians. The primary care teams are distributed evenly throughout the healthcare area based on the number of inhabitants, so that access to healthcare resources can be ensured for everyone. The nurses use the Drago-AP EHR system [18], which includes a module for recording semi-structured nursing assessments by HP as well as for care planning using the NANDA-I, NIC, and NOC SNLs. Thus, after collecting the information available on each HP assessment area, each nurse determines whether a given HP is functional or dysfunctional according to their clinical judgement and, on that basis, establishes the NDs that they deem to be priorities to devise the most suitable care plan while using the NIC and NOC terminologies. This makes it possible to address the care plans of HCCPs in clinical practice, to assess them comprehensively, and to facilitate the diagnostic judgement of priority care needs, as recommended by different authors [19,20].

### 2.3. Study Variables Per Municipality

The sources of information for collecting the data required for this study are the Drago-AP EHR system, the Spanish National Institute of Statistics (SNIS) [21], and the Canary Islands Institute of Statistics (CIIS) [22].

#### 2.3.1. Basic Populations

The variables below were extracted from the SNIS/CIIS:

- Code: A numerical identification code is assigned to each municipality in alphabetical order based on their original names (Figure S1).
- Healthcare areas: metropolitan, north, and south. Each municipality was allocated to one of these areas as per distributions by governmental institutions (Figure S1). As a result, in the study area, 4 municipalities are included in the metropolitan area, 15 in the northern area, and 12 in the southern area.
- Number of inhabitants.
- Mean age (in years) of the municipality inhabitants and mean age of inhabitants with a HCCP profile.
- Percentage of women and percentage of men with a HCCP profile.
- Youth index: expressed as the quotient between the municipality population aged 0–14 years old and the population over 65 years of age (retirement age).
- Crude death rate: deaths per 100,000 inhabitants.
- Percentage of non-Spanish inhabitants.
- Percentage of the population aged 65 and over.
- Percentage of HCCP population and percentage of HCCP population by age range: under 65; between 65 and 79; and aged 80 and over (variables extracted from the Drago-AP EHR system).

#### 2.3.2. Level of Education, in Percentages (Variables Extracted from the SNIS/CIIS)

- No education; compulsory education; secondary education or vocational training; university education.

#### 2.3.3. Financial Variables (Variables Extracted from the SNIS/CIIS)

- Mean gross annual income and mean annual disposable income, in euros.
- Employment: percentages of employed and unemployed population in the previous four-month period; percentage of employed population according to type of work and job position (salaried employees, self-employed, agricultural sector, construction sector, service industries, trade/commerce, and the hospitality sector).

#### 2.3.4. Social Variables (Variables Extracted from the SNIS/CIIS)

- Number of people/families assisted by municipal social services.
- Number of social security benefits granted to the elderly or to persons with a disability/illness.
- Percentage of households by number of cohabiting individuals: households with 1 person; 2 persons; 3 or 4 persons; 5 or more.

#### 2.3.5. Clinical Variables (Variables Extracted from the Drago-AP EHR System)

- Percentage of HCCP population: aged 65 and over by elderly classification (autonomous, frail, dependent); by complexity percentile (high: Pc 95–99.4; very high: ≥99.5); by housebound status; who have been admitted to a hospital in the previous year; who have received medical care from a specialist other than a primary care physician in the previous year; with good dietary habits as recorded in the EHR; who partake in regular physical exercise.
- Mean number of visits per year by the HCCP population to their primary care physician/nurse.

#### 2.3.6. Illness-Related Variables (Variables Extracted from the Drago-AP EHR System)

Data on this type of variable were collected using the percentage of HCCPs experiencing the following health conditions as registered in their EHRs:

- Cardiovascular conditions: high blood pressure; hyperlipidaemia; dysrhythmia; cardiac conduction disorder; ischaemic heart disease; heart failure; valvular heart disease; aortic aneurysm; cerebrovascular accident.
- Endocrinal-metabolic conditions: diabetes mellitus; thyroid disorder; obesity.
- Respiratory conditions: asthma; chronic obstructive pulmonary disease; pneumonia.
- Musculoskeletal conditions: osteoarthritis; arthritis; osteoporosis.
- Nervous system/neurodegenerative conditions: dementia; Parkinson’s; epilepsy; paralysis.
- Mental health conditions: depression; anxiety; schizophrenia; alcohol use disorder; substance abuse; suicidal behaviour or suicide attempt.
- Liver/kidney conditions: chronic renal failure; liver disease.
- Oncological conditions: prior neoplasm; active neoplasm; metastasis; non-Hodgkin lymphoma.
- Other: urinary tract infection; septicaemia; lupus; glaucoma; anaemia.

#### 2.3.7. Pharmacological Treatment (Data Extracted from the Drago-AP EHR System)

- Percentages of the HCCP population with prescribed pharmacotherapy: analgesics; opioids; antidepressants; anxiolytics; hypnotics/sedatives; antipsychotics; anti-dementia drugs.

#### 2.3.8. Dysfunctionality, per HP (Data Extracted from the Drago-AP EHR System)

- Percentages of the HCCP population with a dysfunction recorded in the EHR by each nurse in any of the following eleven assessment areas: (1) health perception/health management; (2) nutrition/metabolism; (3) elimination; (4) physical activity/exercise; (5) sleep/rest; (6) cognition/perception; (7) self-perception/self-concept; (8) role/relationships; (9) sexuality/reproduction; (10) coping/stress tolerance; and (11) values/beliefs.

#### 2.3.9. Nursing Diagnoses (Variables Extracted from the Drago-AP EHR System)

- Mean number of NANDA-I diagnoses assigned to the HCCP population in each municipality; percentages of the HCCP population assigned to each of the 70 NDs included in the study. Consensus as to the inclusion of these NDs was reached by the research team, taking into consideration the objectives of this study as well as the results and conclusions of previous research [15,23]. The 70 NDs considered in this research were those identified as most prevalent in a previous study conducted on the same sample in this healthcare area [15].

### 2.4. Data Collection Procedure

An anonymised database was created using SPSS© v.25.0 statistical software to store all the information collected from each municipality in the healthcare area under study in order to carry out the analyses discussed previously and address the study objectives. Information on population dysfunctionality and data on NDs linked to care needs were processed for geographical representation in the healthcare area using QGIS^®^ v.3.14 software based on the location of each municipality.

### 2.5. Data Analysis

Nominal variables are summarised using the absolute frequency of their categories, while scale variables are expressed as means and standard deviations (SDs) or as medians and interquartile ranges (IQRs), depending on the normality of their distribution. The fulfilment of the normality criterion by the data collected on scale variables was checked using the Kolmogorov–Smirnov test. For bivariate analysis, to explore associations, Pearson or Spearman–Brown correlation coefficients were calculated depending on the normality of the data distribution. The strength of association criteria by Martínez et al. [24] were used: 0–0.25 = none to negligible; 0.26–0.50 = weak; 0.51–0.75 = moderate to strong; 0.76–1.00 = strong to perfect. Bivariate tests for difference analysis were carried out using ANOVA or Kruskal–Wallis statistics, as appropriate. All tests were two-tailed and performed at an alpha significance level of <0.050 using SPSS© (v.25.0.) software from IBM.

### 2.6. Ethical Considerations

The study followed the guidelines set out in the Declaration of Helsinki and was approved by the Research Ethics Committee at the Canary Islands University Hospital Complex under code CHUC_2019_27 (Tenerife, Canary Islands, Spain). This study was carried out in compliance with the Spanish Basic Law 41/2002 of 14 November, regulating patient autonomy, rights, and obligations regarding clinical information and documentation, as well as Regulation (EU) 2016/679 of the European Parliament and of the Council of 27 April 2016 on the protection of natural persons with regard to the processing of personal data. Informed consent was not required, as the main study units were municipalities. This research is epidemiological in nature and does not collect information directly from any patient.

## 3. Results

The basic population characteristics according to studies, i.e., the financial, social, and clinical characteristics of the participating sample, as well as the estimated differences by area, are presented in Table 1. The mean age of HCCPs is significantly higher in the north of the island and lower in the south, as is the prevalence of the general population aged 65 and over. The percentage of HCCPs is also, statistically, significantly higher in the north, an area that appears to be more disadvantaged compared to the other two areas, the metropolitan area and the south. This is due to this area having significantly lower mean gross and disposable incomes, a higher prevalence of unemployed individuals, and a lower frequency of care delivered by medical specialists other than family doctors. On a general population level, the southern area has a lower percentage of women, and the HCCP population is younger. In addition, the prevalence of non-Spanish nationals is significantly higher than in the other two areas, as is the percentage of people working in hospitality. The southern area has the lowest prevalence of households with five or more cohabiting individuals, suggesting that there are fewer people per household in the south than in the rest of the island. However, the highest percentage of hospital admissions in the previous year was found among HCCPs in the south of the island. Finally, the population in the metropolitan area has a more favourable situation. For instance, the prevalence of inhabitants who have completed secondary education or vocational training is higher than in the north or south. The percentage of the population that is salaried is also higher, with a lower prevalence of self-employed workers. In terms of resource use, the prevalence of the care provided to HCCPs by other specialists was significantly higher in the metropolitan area than in the north and south. All the findings described showed statistically significant differences.

Table 2 shows the prevalence of the most frequent conditions among HCCPs, grouped by distribution in areas, the prevalence of pharmacological treatments, and estimated differences. It also shows—by geographical area of each municipality—other less prevalent diseases in which statistically significant differences were identified. Compared to the metropolitan and southern areas, statistically, the northern area exhibits a significantly higher frequency of the following conditions: high blood pressure, osteoarthritis, diabetes mellitus, obesity, urinary tract infection, anaemia, valvular heart disease, schizophrenia, and septicaemia. Similarly, the percentage of anxiolytics and antipsychotics prescribed to HCCPs in the north is higher than in the other areas. However, problems such as osteoporosis and paralysis were significantly more prevalent in the metropolitan area, while ischaemic heart disease was more prevalent in the south.

In order to address objective 2 of the study, Table 3 provides the prevalence of dysfunctionality by HP in the entire territory and by area, as well as the prevalence of care needs by the most frequent NDs, along with estimated differences, and other NDs with significant differences by area.

As shown in Table 3, no significant differences in dysfunctionality were found for each HP in the geographical areas classified as metropolitan, north, or south. However, a higher average number of NDs among HCCPs was identified in the north, with significant differences compared to the other two areas. There is also a higher prevalence of the following thirteen NDs in the north: Ineffective protection (00043), Risk for falls (00155), Impaired urinary elimination (00016), Risk for infection (00004), Bathing self-care deficit (00108), Impaired physical mobility (00085), Dressing self-care deficit (00109), Constipation (00011), Impaired tissue integrity (00044), Decreased diversional activity engagement (00097), Feeding self-care deficit (00102), Toileting self-care deficit (00110), and Risk-prone health behavior (00188). With significant differences, a higher prevalence of Noncompliance (00079) and Risk for bleeding (00206) was found in the southern area, while the prevalence of Ineffective health maintenance (00099) was higher in the metropolitan area. The correlations identified between the prevalences of dysfunctionality and NDs in the community and each sociodemographic, financial, and clinical characteristic (objective 3) are described in Table 4 and Table S1 (Supplementary Materials), respectively.

Table 4 shows a high number of significant correlations between dysfunctionality by HP and the different population characteristics. However, according to the criteria established by Martínez et al. [24], moderate/strong associations were identified between greater dysfunctionality in HP 3 (Elimination) and a higher percentage of older HCCPs on the one hand and a higher frequency of prescribed analgesics on the other. Moderate/strong associations were also observed between a higher percentage of dysfunctionality in HP 4 (Physical activity/Exercise) and a higher prevalence of the HCCP population, as well as between HP 4 and a higher prevalence of the HCCP population aged 80 years or older. Finally, a moderate/strong correlation was found between a higher prevalence of dysfunctionality in HP 5 (Sleep/Rest) and a higher population frequency of arthritis among HCCPs.

Table S1 shows that a group of ten NDs is moderately/strongly correlated with at least ten of the variables under study. These NDs are Dressing self-care deficit (00109), Toileting self-care deficit (00110), Chronic pain (00133), Bathing self-care deficit (00108), Impaired memory (00131), Risk for falls (00155), Functional urinary incontinence (00020), Imbalanced nutrition: less than body requirements (00002), Chronic confusion (00129), and Risk for pressure ulcer (00249). This table also shows that a group of seven basic population characteristics were moderately/strongly associated with more than 20% of all NDs, namely: percentage of population aged 65 or over; mean age of inhabitants in the municipality; percentage of the HCCP population; mean age of the HCCP inhabitants; percentage of the HCCP population aged 80 or over; percentage of the HCCP population under 65; and crude mortality rate.

In order to address objective 4 of the study, the Supplementary Materials includes the findings—geographically represented by municipality—on the prevalence of population dysfunctionality (by HP) (Figures S2–S12) and the most frequently identified care needs (NDs) among HCCPs (Figures S13–S37).

Taking the percentages of dysfunctionality pattern by pattern and municipality by municipality (Figures S2–S12), the five municipalities with the highest prevalence are located in the northern and southern areas in 96.4% of cases. Two of these municipalities—located in the south (coded as Nos. 3 and 31 in Figure S1)—are among the five with the highest prevalence of dysfunctionality in ten out of the eleven HPs. In addition, four municipalities in the north (Nos. 17, 29, 19, and 15 in Figure S1) are among those with the highest prevalence of dysfunctionality, at least in 36.4% of HPs.

Significant differences were found in the prevalence of community care needs among HCCPs (identified using DEs) by the area in which the municipality was located (metropolitan, north, or south). As a result, the ND Ineffective protection (00043) (Figure S18) was identified in at least 25% of HCCPs in 93.3% of the municipalities in the north. Risk for falls (00155) (Figure S20) was diagnosed in at least 20% of HCCPs in half of the island’s municipalities, 75% of which are located in the north. Impaired urinary elimination (00016) (Figure S21) was present in at least 20% of HCCPs in eleven municipalities on the island (35%), all in the north. Eight out of the ten municipalities with the highest prevalence of Risk for infection (00004) (Figure S26) were also in the north.

In contrast, the geographical distribution of Noncompliance (00079) (Figure S30) shows a higher prevalence in municipalities in the south. Thus, 70% of the ten municipalities with the highest frequency of noncompliance among HCCPs are located in the south. Of the 15 municipalities with the highest prevalence of Bathing self-care deficit (00108) (Figure S31)—almost half of the municipalities in the entire healthcare area—80% are located in the north. Similarly, the NDs Impaired physical mobility (00085) (Figure S34) and Dressing self-care deficit (00109) (Figure S35) are more prevalent in the north. Of the ten municipalities with the highest frequency of Impaired physical mobility (00085), nine are in the north, as are thirteen (86.7%) of the fifteen municipalities with the highest frequency of Dressing self-care deficit (00109).

Finally, the only ND with a significantly higher prevalence in the metropolitan area is Ineffective health maintenance (00099) (Figure S36). However, its mean prevalence among HCCPs is close to that of the northern area (as shown in Table 3), but notably higher, in both cases, than the frequency with which this ND is identified in the southern municipalities.

Although two municipalities in the entire healthcare area (Nos. 3 and 31, Figure S1) were identified as frequently dysfunctional in almost all HPs, only one of these municipalities (No. 31) had the highest prevalence of a single ND, i.e., Risk for infection (00004) (Figure S26).

## 4. Discussion

The healthcare area under study is part of the Spanish Autonomous Community of the Canary Islands, one of the most disadvantaged at the national level, according to the latest report published in Spain by the European Anti-Poverty Network [25]. The Canary Islands are among the southernmost and westernmost communities in Spain (being the south-western border of the European Union), showing high rates of social and economic inequality when compared to the north and east. Previous studies highlight the strong relationship between social inequalities in the Canary Islands and its population care needs, as identified using NDs [26].

Within the territory under study, the island of Tenerife, there are significant social and economic differences between municipalities depending on whether they are part of the metropolitan, the northern, or the southern area (Table 1). Firstly, municipalities in the north have an older population, which is more in consonance with the HCCP profile. In addition, the north has a higher prevalence of HCCPs, lower mean income, higher unemployment rates, and less frequent assistance provided to HCCPs by specialists other than primary and community care specialists. The latter is not necessarily a negative aspect, as it could be an indication—subject to further supporting evidence—of more and/or better monitoring of HCCPs by primary care teams, as opposed to a greater number of medical referrals.

Secondly, the population profile of the southern area features a larger proportion of non-Spanish nationals, fewer women, and a higher prevalence of the hospitality sector, as well as a higher proportion of households with fewer cohabiting individuals. These characteristics are common to the entire autonomous community, where tourism traditionally plays a crucial role in economic development and has been a factor of great interest in explaining territorial imbalances [27]. The southern area has a younger HCCP profile but a higher frequency of hospital admissions, which could be indicative of poorer management of disease processes by primary care or poorer adherence to treatments.

Thirdly, in the metropolitan area, which generally has a larger population, the proportion of HCCPs attended to by specialists outside primary care is significantly higher. This could be interpreted as a potentially greater demand for healthcare than in the north or south, as there is a larger population to serve, which could lead to more referrals and more difficulties in the continuity of care. In population terms, the metropolitan area has a higher proportion of individuals with advanced education (high school, vocational training, or university studies), higher employment rates, a greater number of salaried employees, and fewer self-employed workers. These characteristics are a priori more favourable for providing and maintaining good-quality health care [28].

The geographical distribution of health conditions among HCCPs was significantly higher for a large group of them in the northern municipalities (Table 2). This is consistent with the estimated all-cause mortality risks by municipality, which are higher among those in the north [29]. This finding characterises HCCPs in the north as communities with higher morbidity and mortality than the rest of the population. These significantly more prevalent conditions include serious mental health conditions such as schizophrenia and, likely correlated, a higher frequency of prescribed pharmacological treatments such as anxiolytics and antipsychotics. It is well known that a more urban or rural nature of a given population is not associated with a higher prevalence of schizophrenia and that less developed countries have a lower prevalence of this mental health condition [30]. We failed to find studies analysing these issues among HCCPs in our context. However, as a result of this research, we did observe much higher frequencies of chronic conditions that have been reported as the most prevalent in the general population by the Estrategia para el Abordaje de la Cronicidad in the Canary Islands [31], namely depression, diabetes mellitus, ischaemic heart disease, chronic obstructive pulmonary disease, anxiety, and thyroid disorder. These findings are to be expected, as we are dealing with the most clinically complex segment of chronically ill patients.

The percentages of dysfunctionality among the HCCP population showed no significant differences by area (Table 3). Nonetheless, the levels of dysfunctionality reported in the EHRs were higher in the north in six out of the eleven HPs, especially in patterns assessing the psychosocial dimension of individuals (HPs 1 and 7–11). The northern area also has a larger population engaged in agriculture than the metropolitan and southern areas (Table 1). This, coupled with the aforementioned lower income and higher prevalence of mental health treatments and conditions such as schizophrenia among HCCPs, results in greater vulnerability in terms of health needs among poorer populations and rural contexts, which is in line with previous studies [32]. The most dysfunctional HPs across the entire healthcare area are: Cognition/Perception (HP 6), with a higher prevalence in the metropolitan area, i.e., almost 7 out of 10 HCCPs; Self-perception/Self-concept (HP 7); Physical activity/Exercise (HP 4); and Nutrition/Metabolism (HP 2). More than half of the population in all areas has all the dysfunctional HPs. The latter two HPs also have higher frequencies in the metropolitan area.

The distribution of care needs, which is higher for thirteen NDs in the north (Table 3), indicates a significant functional vulnerability profile, in line with what has been discussed above about this geographical area. The higher frequency of the ND Noncompliance (00079) in the south could be linked to the aforementioned higher percentage of prior hospital admissions among HCCPs. Similarly, the higher prevalence of Ineffective health maintenance (00099) in the metropolitan area could be related to the higher proportion of HCCPs seen by non-primary care specialists and to the possible difficulties in continuity of care due to the high demand for care and pressure on the public health system caused by the greater population numbers, as mentioned above.

All these findings may highlight the clinical value of standardised nursing languages in EHRs as important descriptors of health conditions requiring professional care and their predictive power on the use of healthcare resources, as demonstrated in other research [33]. The results of our study provide a response to the research needs in the field of NDs [34], in this case from a community perspective.

The correlations identified—moderate/strong associations (Table 4) between higher prevalences of dysfunctionality in HPs 3 and 4 and higher frequencies of the older HCCP population—were to be expected. High clinical complexity and greater dysfunctionality in daily life, although not exclusive to older individuals, are more common in this population profile [35]. Therefore, the proven relationship between higher dysfunctionality in HP 4 and the higher proportion of HCCPs in the municipality is also consistent. In addition, a moderate/strong association was found between a higher proportion of HCCPs with dysfunctional HP 3 and a higher frequency of prescribed analgesics in the municipalities under study. A priori, pending further studies on the potential reasons for this association, this finding does not seem to have a clear explanation. However, the equally moderate/strong correlation between a higher percentage of dysfunctional HP 5 and a higher prevalence of arthritis could have a plausible explanation, as arthritis, being a chronic health problem, can contribute to disturbed sleep [36].

In Table S1, the ten NDs that are moderately/strongly correlated with the greatest number of sociodemographic variables (especially those that take age into consideration) portray an HCCP profile of an older person with functional problems in self-care in a range of activities of daily living, with chronic pain, cognitive, urinary, and nutritional impairment, as well as potential risks of falling and pressure ulcers. This reinforces the previously stated idea that there are NDs representative of these populations, which establishes priority community care needs among HCCPs.

Table S1 also shows moderate/strong correlations between the presence of certain NDs and socio-economic variables reflecting social inequality. For instance, it was found that a higher prevalence of the uneducated population indicated a higher frequency of the ND Acute pain (00132), as reported by other authors [37]. It was also observed that a lower mean income indicated a higher prevalence of the ND Chronic pain (00133), which has also been confirmed in the literature [38]. In our research, we found that a lower mean income indicated a higher prevalence of the ND Readiness for enhanced health management [00162], which might suggest that, despite inequalities, the willingness of these individuals works in their favour despite the health-related difficulties encountered.

It is also important to note the higher prevalence of several NDs in populations with a higher frequency of prescribed drugs. For example, in municipalities with a higher prevalence of antidepressants, a higher proportion of the following NDs was observed: Risk for falls (00155), Impaired urinary elimination (00016), Imbalanced nutrition: less than body requirements (00002), Insomnia (00095), and Impaired memory (00131). Previous studies identify significant correlations between depression and certain NDs in similar populations [39]. Another group of drugs, antipsychotics, was moderately/strongly associated with a greater prevalence of NDs such as Dressing self-care deficit (00109), Decreased diversional activity engagement (00097), Chronic confusion (00129), and Disturbed thought processes (00130). The potential adverse effects of these drugs are known to negatively influence the quality of life of patients [40].

As expected, in municipalities with a higher percentage of prescribed analgesics, a higher prevalence of Chronic pain (00133), Anxiety (00146), Insomnia (00095), and Grieving (00136) was also observed. The association between the prescription of analgesics and issues such as anxiety and depressive symptoms and their management has been studied previously, including in the context of bereavement [41]. It is well known that in the management of HCCPs, especially in the case of elderly individuals, professionals must pay special attention to medicine reconciliation by reviewing potentially inappropriate prescription drugs and their impact on the health and quality of life of these patients [42].

Limitations in wordcount preclude a detailed account of each correlation between NDs and sample characteristics. Therefore, having highlighted some of the most important findings, we invite readers to take a closer look at the remaining data available in Table S1 as Supplementary Materials.

Figures S2–S37 display the prevalences of dysfunctionality (HPs) and community care needs (NDs), municipality by municipality, thus demonstrating the hypothesised heterogeneity of their distribution across geographical areas, with the north being clearly disadvantaged. Health inequalities in the Spanish population are linked to social determinants such as place of residence, occupation, level of education, and socioeconomic status [43], all of which were explored in this study.

It should also be noted that the geographical characteristics of one specific municipality are among those with the highest prevalence of dysfunctionality in ten of the eleven HPs (no. 31, Figure S1) and that it also has a high prevalence of several care needs. This municipality is located at 1400 metres above sea level, being one of the Spanish towns at the highest altitude. It is known that altitude is associated with the occurrence of both acute and chronic health conditions [44,45], which could contribute to the greater population dysfunctionality and the presence of certain care needs identified in our study in this municipality. However, further studies are needed in our context to confirm this relationship between dysfunctionality, care needs, and the altitude at which each population group lives.

This study has several limitations. Firstly, the unavailability of previous epidemiological studies analysing dysfunctionality and population needs from a community approach using standardised nursing languages. As such, our study could serve as a starting point for future research, focusing on a highly demanding population in terms of healthcare, such as HCCPs. Secondly, our analysis focuses on a limited number of municipalities that make up a relatively small sample. It would therefore be of great interest to replicate our study in new populations, whether in the same geographical context or in others, to try to confirm or refute each of our findings. However, all HCCPs in each municipality in the study area were included in our analyses, enabling us to provide a complete picture of the entire population profile. Thirdly, an observational, descriptive, cross-sectional method was used, which does not allow for cause–effect testing, as this would require a different design and follow-up of the population groups in a cohort study. However, our research team has new projects underway in the form of prospective studies monitoring a larger cohort of municipalities with a HCCP population throughout the entire autonomous community of the Canary Islands. Finally, this study was carried out using statistical data from institutional sources as well as clinical data extracted from EHRs. This is not an easily surmountable limitation, as this is a population-based epidemiological study, which, by its very nature, requires extracting data from a wide range of available records.

## 5. Conclusions

Correlations were found between the prevalence of dysfunctionality and care needs and several socio-demographic, financial, and clinical characteristics in each municipality. Differences were also identified between the northern, southern, and metropolitan areas, with the northern area being the most disadvantaged. Furthermore, the community distribution by municipality of dysfunctionality (by HP) and care needs (per ND reported in EHRs) were geographically represented through prevalence maps.

In light of the results obtained, we believe that our study contributes to advancing the epidemiological knowledge of the population needs of HCCPs through the use of standardised nursing terminology while facilitating the implementation of new studies in the same line of research. These studies could explore a greater number of municipalities and healthcare areas and form a population cohort for follow-up and/or replicating our study in other geographical and clinical contexts and/or with different patient profiles.

## Figures and Tables

**Table 1:** Characteristics of the municipalities in the healthcare area under study, their distribution by area, and estimated differences.

Characteristics of the Municipalities	Mean (SD) or Median (IQR) ^a^				*p*-Value	95% CIs ^b^ or Average Ranges		
	Area	Metropolitan	North	South		Metropolitan	North	South
Basic population characteristics								
Population (no. of inhabitants) ^c^	14,987 (24,626)	87,800 (183,032.75)	9114 (19,492)	21,413.50 (37,404)	0.156	23.25	13.53	16.67
Age (years)	42.92 (2.32)	42.98 (0.83)	43.72 (1.89)	41.90 (2.83)	0.128	41.66–44.29	42.67–44.77	40.11–43.70
Age of HCCPs ^d^ (years)	73.41 (1.45)	73.68 (1.06)	74.07 (1.04)	72.49 (1.58)	0.012 *	71.99–75.37	73.50–74.65	71.48–73.49
Women (%)	50.19 (1.27)	51.03 (1.53)	50.58 (0.76)	49.42 (1.38)	0.016 *	48.60–53.47	50.16–51.00	48.54–50.29
Female HCCPs (%)	56.18 (2.86)	58.00 (3.52)	55.93 (2.92)	55.89 (2.58)	0.407	52.40–63.60	54.31–57.54	54.26–57.53
Youth index	12.90 (1.52)	12.85 (0.69)	12.54 (1.38)	13.37 (1.83)	0.384	11.75–13.95	11.78–13.31	12.21–14.53
Crude death rate per 100,000 inhabitants	807.67 (269.43)	742.90 (156.13)	913.81 (251.77)	696.58 (283.39)	0.097	494.46–991.34	774.38–1053.23	516.53–876.64
Non-Spanish nationals (%) ^c^	6.85 (11.45)	6.24 (3.46)	5.71 (3.32)	19.57 (31.10)	0.005 *	11.00	12.00	22.67
Population aged 65 and over (%)	17.82 (4.14)	16.73 (2.10)	19.69 (3.73)	15.85 (4.29)	0.043 *	13.39–20.07	17.62–21.76	13.13–18.58
HCCP population (%)	5.94 (1.65)	5.62 (1.00)	6.71 (1.03)	5.07 (2.03)	0.028 *	4.02–7.22	6.15–7.28	3.79–6.36
HCCP population per age range (%):								
<65 years	21.86 (4.18)	21.45 (3.76)	19.86 (2.87)	24.50 (4.48)	0.011 *	15.47–27.43	18.27–21.45	21.65–27.35
65–79 years	43.69 (2.03)	43.63 (1.06)	44.09 (1.56)	43.22 (2.72)	0.558	41.94–45.31	43.22–44.95	41.49–44.95
≥80 years	34.45 (4.51)	34.95 (2.82)	36.05 (3.07)	32.28 (5.70)	0.187	30.47–39.43	34.35–37.75	28.66–35.90
Level of education								
No education (%)	9.09 (4.90)	4.65 (1.54)	10.19 (4.98)	9.19 (4.94)	0.131	2.20–7.10	7.43–12.95	6.06–12.33
Compulsory education (%)	25.82 (5.91)	17.72 (3.78)	27.27 (3.78)	26.71 (6.76)	0.008 *	11.71–23.73	25.18–29.37	22.42–31.01
Secondary education or vocational training (%)	42.37 (4.66)	47.49 (4.37)	40.31 (3.46)	43.24 (4.75)	0.012 *	40.53–54.44	38.40–42.23	40.22–46.25
University education (%)	22.72 (7.47)	30.15 (0.81)	22.23 (7.00)	20.86 (8.13)	0.089	28.85–31.44	18.36–26.09	15.69–26.02
Financial characteristics								
Mean gross annual income (euros) ^c^	20,295 (5682)	27,476 (5058.50)	18,862 (4392)	19,938.50 (3607.50)	0.010 *	28.75	13.63	14.71
Mean disposable annual income (euros) ^c^	17,364 (4251)	22,537 (3518.00)	16,389 (3080)	17,105.50 (2520.00)	0.012 *	28.50	13.67	14.75
Employed population in the previous four-month period (%) ^c^	22.58 (11.49)	39.49 (30.17)	21.52 (4.45)	25.31 (11.87)	0.032 *	23.50	11.87	18.67
Unemployed population in the previous four-month period (%) ^c^	12.61 (1.98)	11.10 (3.57)	12.88 (0.98)	11.30 (2.21)	0.023 *	9.00	20.47	12.75
Salaried employees (%)	76.13 (6.10)	81.30 (10.02)	73.33 (3.82)	77.90 (5.62)	0.023 *	65.36–97.25	71.21–75.45	74.33–81.47
Self-employed (%)	23.87 (6.10)	18.70 (10.02)	26.67 (3.82)	22.10 (5.62)	0.023 *	2.75–34.64	24.55–28.79	18.53–25.67
Agricultural sector (%) ^c^	4.24 (6.64)	1.43 (3.78)	5.66 (12.58)	4.59 (7.03)	0.060	7.00	19.00	15.25
Construction sector (%) ^c^	8.37 (4.82)	7.69 (4.58)	10.07 (7.65)	6.94 (3.73)	0.108	11.75	19.53	13.00
Service industries (%) ^c^	76.68 (10.29)	80.44 (16.94)	75.57 (12.69)	80.24 (13.00)	0.518	17.75	14.07	17.83
Trade/Commerce (%)	19.62 (6.92)	20.08 (4.18)	18.98 (6.46)	20.27 (8.47)	0.888	13.42–26.74	15.40–22.56	14.89–25.65
Hospitality sector (%) ^c^	11.99 (13.99)	6.35 (3.15)	10.45 (8.03)	21.13 (24.73)	0.004 *	3.75	15.13	21.17
Social characteristics								
No. of people assisted by SS ^c,e^	4115 (6904)	8759.50 (10502.25)	4115 (6925)	3617.00 (6225.50)	0.605	20.25	15.33	15.42
No. of families assisted by SS ^c^	942.00 (1469.25)	2267.00 (10164.25)	950 (1344)	832 (2947)	0.844	17.00	14.60	16.18
No. of social security benefits granted to the elderly ^c^	1380 (2920)	3708.50 (8112.00)	1380 (2929)	1114.00 (2602.25)	0.558	20.25	16.00	14.58
No. of social security benefits granted to persons with a disability/illness ^c^	756 (1249)	1404.50 (1734.00)	756 (1415)	391.50 (1109.50)	0.530	18.50	17.13	13.75
Households with:								
1 person (%)	21.15 (5.25)	18.56 (4.64)	20.06 (4.66)	23.36 (5.66)	0.153	11.17–25.95	17.48–22.64	19.77–26.96
2 persons (%)	29.03 (4.24)	29.46 (1.33)	26.97 (2.88)	31.46 (5.08)	0.018 *	27.35–31.58	25.38–28.57	28.24–34.69
3–4 persons (%)	43.70 (5.18)	43.63 (2.25)	45.62 (5.30)	41.32 (4.97)	0.097	40.06–47.21	42.69–48.56	38.16–44.48
≥5 persons (%)	6.12 (3.67)	8.35 (1.61)	7.34 (3.69)	3.86 (3.06)	0.016 *	5.78–10.91	5.30–9.39	1.91–5.80
Clinical characteristics								
HCCP population:								
≥65 years, autonomous (%)	48.49 (5.99)	52.25 (3.49)	46.32 (4.69)	49.95 (7.26)	0.118	46.70–57.80	43.72–48.92	45.34–54.56
≥65 years, frail (%)	35.06 (5.38)	31.60 (4.78)	37.33 (3.75)	33.37 (6.36)	0.058	24.00–39.20	35.26–39.41	29.33–37.41
≥65 years, dependent (%)	16.46 (3.01)	16.20 (2.34)	16.36 (2.88)	16.67 (3.55)	0.953	12.47–19.93	14.77–17.95	14.41–18.92
Very high complexity (Pc ^f^ ≥ 99.5) (%)	11.05 (1.79)	10.20 (0.77)	11.65 (1.36)	10.58 (2.28)	0.179	8.97–11.43	10.90–12.41	9.13–12.02
Housebound (%)	15.34 (3.20)	17.10 (0.95)	15.94 (0.74)	14.01 (1.04)	0.149	14.07–20.13	14.35–17.53	11.72–16.29
Admitted to a hospital in the previous year (%) ^c^	7.50 (14.20)	8.30 (12.65)	6.00 (2.20)	19.20 (1.58)	<0.001 *	12.50	9.47	25.33
Seen by a specialist other than a family doctor in the previous year (%) ^c^	1.90 (4.20)	4.20 (1.85)	0.30 (2.50)	1.90 (7.80)	0.016 *	22.88	11.90	18.83
Good dietary habits (%)	65.13 (20.03)	75.95 (8.69)	62.23 (25.07)	65.15 (14.83)	0.492	62.12–89.78	48.34–76.11	55.73–74.57
Regular physical exercise (%)	22.19 (10.20)	31.18 (6.83)	22.00 (10.19)	19.41 (10.05)	0.135	20.32–42.04	16.12–27.89	13.02–25.80
Mean no. of visits/year to their family doctor	9.22 (0.78)	8.90 (0.43)	9.05 (0.62)	9.53 (0.97)	0.205	8.21–9.59	8.71–9.40	8.91–10.14
Mean no. of visits/year to their primary care nurse	5.77 (1.12)	5.32 (0.50)	6.14 (1.25)	5.45 (0.99)	0.200	4.53–6.11	5.45–6.83	4.82–6.08

^a^ Mean (SD) or Median (IQR): mean (standard deviation) or median (interquartile range). ^b^ 95% CIs: 95% confidence intervals. ^c^ Non-normally distributed variable. ^d^ HCCPs: highly complex chronic patients. ^e^ SS: Social Services. ^f^ Pc: percentile. * Statistically significant *p*-value.

**Table 2:** Prevalence (%) of conditions and prescribed pharmacological treatments among highly complex chronic patients, distributed by area and difference tests.

Prevalence of Conditions and Pharmacological Treatments (%)	Mean (SD) or Median (IQR) ^a^				*p*-Value	95% CIs ^b^ or Average Ranges		
	Area	Metropolitan	North	South		Metropolitan	North	South
Conditions								
High blood pressure	87.20 (2.63)	85.30 (1.89)	88.67 (2.73)	86.82 (2.07)	0.031 *	82.30–88.31	87.16–90.18	85.50–88.13
Hyperlipidaemia	80.45 (3.03)	80.05 (2.15)	81.29 (2.93)	79.54 (3.30)	0.329	76.63–83.47	79.66–82.91	77.44–81.64
Osteoarthritis	68.10 (4.58)	69.78 (2.58)	69.87 (3.74)	65.32 (4.88)	0.021 *	65.66–73.89	67.80–71.95	62.22–68.42
Diabetes mellitus	57.61 (4.25)	53.80 (3.64)	60.37 (3.23)	55.43 (3.37)	<0.001 *	48.01–59.59	58.58–62.16	53.28–57.57
Dysrhythmia ^c^	40.60 (2.50)	39.35 (2.40)	40.90 (5.70)	40.20 (5.40)	0.081	11.13	19.73	12.96
Obesity ^c^	38.70 (6.70)	39.00 (3.35)	42.40 (9.30)	36.05 (6.73)	0.005 *	16.13	21.03	9.67
Depression	33.35 (3.29)	34.63 (3.28)	33.76 (2.78)	32.41 (3.88)	0.418	29.40–39.85	32.22–35.30	29.95–34.87
Anxiety	32.42 (5.35)	31.88 (3.51)	33.71 (6.74)	30.98 (3.48)	0.422	26.30–37.45	29.98–37.45	28.76–33.19
Asthma	30.25 (3.78)	29.58 (1.07)	31.03 (4.78)	29.49 (2.81)	0.550	27.87–31.28	28.38–33.68	27.71–31.28
Thyroid disorder ^c^	29.40 (2.10)	29.10 (0.75)	29.90 (2.50)	28.90 (4.18)	0.516	15.75	17.83	13.79
Chronic renal failure	28.02 (3.59)	26.93 (1.28)	26.93 (3.93)	29.75 (3.13)	0.101	24.89–28.96	24.76–29.11	27.76–31.74
Cardiac conduction disorder	28.00 (4.78)	28.63 (1.66)	29.00 (4.14)	26.53 (5.80)	0.409	23.35–33.90	26.71–31.29	22.85–30.22
Urinary tract infection	24.58 (2.85)	23.00 (1.49)	26.13 (3.11)	23.16 (1.69)	0.009 *	20.64–25.36	24.41–27.86	22.08–24.23
Ischaemic heart disease	24.29 (2.72)	24.48 (3.18)	23.00 (2.72)	25.85 (1.76)	0.020 *	19.41–29.54	21.49–24.51	24.74–26.97
Chronic obstructive pulmonary disease	24.01 (4.21)	22.43 (1.47)	22.97 (4.41)	25.83 (4.13)	0.155	20.09–24.76	20.53–25.41	23.21–28.46
Heart failure	24.00 (2.81)	23.48 (2.43)	24.44 (3.17)	23.63 (2.59)	0.712	19.61–27.34	22.69–26.19	21.98–25.27
Prior neoplasm	20.07 (2.09)	21.13 (1.21)	19.91 (2.32)	19.93 (2.05)	0.572	19.20–23.05	18.62–21.22	19.31–20.84
Other conditions with statistically significant differences								
Anaemia	17.15 (2.22)	16.35 (1.11)	18.18 (2.37)	16.13 (1.77)	0.038 *	14.59–18.12	16.87–19.49	15.01–17.26
Valvular heart disease	16.42 (3.46)	18.30 (2.10)	18.39 (2.96)	13.33 (1.82)	<0.001 *	14.97–21.64	16.75–20.02	12.17–14.48
Osteoporosis	14.46 (2.00)	16.53 (1.80)	14.60 (1.90)	13.60 (1.74)	0.032 *	13.66–19.39	13.55–15.65	12.49–14.71
Paralysis	4.52 (1.39)	5.13 (1.30)	4.98 (1.52)	3.75 (0.89)	0.041 *	3.07–7.19	4.14–5.82	3.19–4.32
Schizophrenia	3.50 (1.04)	2.35 (0.40)	3.97 (0.97)	3.28 (0.93)	0.009 *	1.71–2.99	3.44–4.51	2.69–3.88
Septicaemia	1.10 (0.70)	1.20 (0.55)	1.50 (1.10)	0.80 (0.35)	0.002 *	17.25	21.33	8.92
Pharmacological treatments								
Analgesics	56.14 (4.85)	53.78 (2.15)	56.21 (3.05)	56.83 (6.97)	0.567	50.35–57.20	54.52–57.90	52.40–61.25
Antidepressants	34.85 (3.12)	33.95 (4.35)	35.88 (2.64)	33.86 (3.13)	0.209	27.03–40.88	34.42–37.34	31.87–35.85
Anxiolytics	27.21 (3.43)	24.60 (5.14)	28.67 (2.76)	26.25 (2.97)	0.045 *	16.42–32.78	27.15–30.20	24.36–28.14
Opioids	21.40 (3.20)	21.10 (0.21)	20.93 (3.17)	22.09 (3.79)	0.649	20.76–21.44	19.18–22.69	19.69–24.50
Hypnotics/sedatives	15.21 (2.91)	13.63 (2.07)	16.03 (3.33)	14.72 (2.41)	0.267	10.33–16.92	14.18–17.87	13.18–16.25
Antipsychotics	10.97 (2.46)	9.48 (1.13)	12.05 (1.81)	10.11 (2.96)	0.048 *	7.68–11.27	11.05–13.05	8.23–11.99
Anti-dementia drugs	6.47 (1.85)	6.35 (0.87)	6.58 (1.59)	6.37 (2.43)	0.924	4.96–7.74	5.70–7.46	4.82–7.91

^a^ Mean (SD) or Median (IQR): mean (standard deviation) or median (interquartile range). ^b^ 95% CIs: 95% confidence intervals. ^c^ Non-normally distributed variable. * Statistically significant *p*-value.

**Table 3:** Prevalence data (%) in a highly complex chronic patient population: dysfunctionality by Gordon’s health patterns and care needs by NANDA-I diagnoses per healthcare sector under study, distributed by difference tests.

Dysfunctionality and Care Needs (%)	Mean (SD) or Median (IQR) ^a^				*p*-Value	95% CIs ^b^ or Average Ranges		
	Area	Metropolitan	North	South		Metropolitan	North	South
Gordon’s health patterns								
1. Health perception/Health management	51.86 (12.78)	52.05 (5.31)	52.40 (9.50)	51.12 (17.94)	0.969	43.60–60.51	47.14–57.66	39.72–62.52
2. Nutrition/Metabolism ^c^	54.00 (8.70)	56.50 (9.38)	53.50 (8.00)	52.95 (14.05)	0.452	21.13	15.80	14.54
3. Elimination	45.17 (9.55)	50.00 (6.64)	46.31 (6.60)	42.13 (12.68)	0.303	39.43–60.57	42.65–49.96	34.08–50.19
4. Physical activity/Exercise	54.17 (9.07)	57.38 (1.76)	56.02 (7.34)	50.80 (11.59)	0.257	54.58–60.17	51.95–60.09	43.44–58.16
5. Sleep/Rest	46.10 (9.26)	45.78 (7.86)	44.87 (8.43)	47.74 (11.03)	0.737	33.28–58.28	40.20–49.54	40.73–54.75
6. Cognition/Perception	65.18 (9.07)	67.48 (3.87)	66.29 (8.81)	63.02 (10.63)	0.575	61.32–73.63	61.42–71.17	56.26–69.77
7. Self-perception/Self-concept	55.93 (12.62)	53.28 (12.20)	57.65 (12.80)	54.66 (13.29)	0.761	33.86–72.69	50.56–64.74	46.21–63.10
8. Role/Relationships	42.84 (13.03)	41.10 (10.84)	44.31 (11.88)	41.58 (15.67)	0.839	23.86–58.34	37.73–50.89	31.63–51.54
9. Sexuality/Reproduction	12.92 (6.17)	11.75 (3.75)	13.83 (6.77)	12.18 (6.29)	0.740	5.78–17.72	10.08–17.58	8.19–16.18
10. Coping/Stress tolerance	45.49 (15.32)	41.55 (14.86)	47.15 (14.04)	44.72 (17.85)	0.801	17.90–65.20	39.38–54.93	33.38–56.06
11. Values/Beliefs ^c^	14.80 (12.80)	10.55 (10.83)	16.70 (13.40)	14.55 (11.78)	0.587	11.63	16.67	16.63
Nursing diagnoses (NANDA-I code)								
Mean number of diagnoses	7.44 (1.45)	6.74 (0.55)	8.14 (1.50)	6.80 (1.23)	0.028 *	5.86–7.61	7.31–8.97	6.01–7.58
Readiness for enhanced health management (00162) ^c^	78.20 (8.20)	75.20 (8.75)	79.30 (6.10)	78.70 (22.98)	0.189	8.63	17.97	16.00
Willingness to improve immunization status (00186)	44.53 (11.29)	40.50 (0.80)	48.19 (12.67)	41.30 (10.36)	0.221	39.23–41.77	41.17–55.20	34.72–47.88
Impaired skin integrity (00046)	43.22 (9.73)	40.58 (4.47)	46.97 (10.29)	39.41 (8.95)	0.110	33.46–47.69	41.28–52.67	33.72–45.10
Acute pain (00132)	42.06 (11.39)	38.08 (3.15)	45.15 (12.08)	39.53 (11.83)	0.348	33.06–43.09	38.46–51.84	32.02–47.05
Impaired comfort (00214)	27.72 (9.64)	25.38 (5.12)	24.32 (6.43)	32.74 (12.19)	0.160	17.23–33.52	20.76–27.88	25.00–40.49
Ineffective protection (00043) ^c^	22.90 (22.60)	19.30 (7.93)	27.20 (10.30)	4.75 (2.73)	<0.001 *	16.50	23.20	6.83
Ineffective breathing pattern (00032)	20.88 (6.24)	18.55 (2.57)	23.37 (7.06)	18.55 (4.98)	0.097	14.47–22.64	19.46–27.27	15.39–21.71
Risk for falls (00155)	20.31 (6.27)	19.50 (1.80)	23.18 (5.74)	17.00 (6.41)	0.032 *	16.63–22.37	20.00–26.36	12.93–21.07
Impaired urinary elimination (00016)	18.85 (6.00)	16.13 (2.34)	22.74 (5.78)	14.90 (3.72)	0.001 *	12.40–19.85	19.54–25.94	12.53–17.27
Risk for unstable blood glucose level (00179) ^c^	18.40 (6.90)	18.35 (3.93)	19.50 (6.90)	16.00 (6.10)	0.236	13.25	18.87	13.33
Nutritional imbalance: excess (00001)	15.54 (5.10)	16.75 (4.21)	15.91 (5.69)	14.67 (4.84)	0.735	10.05–23.45	12.76–19.06	11.59–17.74
Chronic pain (00133)	14.41 (6.98)	10.55 (2.99)	15.43 (5.32)	14.42 (9.35)	0.477	5.80–15.30	12.49–18.38	8.48–20.36
Ineffective health management (00078) ^c^	14.30 (8.30)	12.15 (4.28)	16.40 (8.70)	12.00 (8.15)	0.420	11.88	18.00	14.88
Risk for infection (00004) ^c^	14.20 (8.30)	13.65 (3.48)	17.00 (5.40)	10.55 (7.30)	0.020 *	15.00	20.50	10.71
Risk for injury (00035) ^c^	13.20 (9.90)	10.25 (6.45)	13.60 (14.20)	12.75 (9.9)	0.242	10.75	18.57	14.54
Anxiety (00146)	13.17 (5.11)	9.98 (3.07)	13.83 (3.65)	13.43 (6.89)	0.413	5.09–14.86	11.81–15.85	9.05–17.80
Health-generating behaviors (00084)	13.10 (6.09)	10.55 (3.07)	13.21 (6.22)	13.81 (6.82)	0.663	5.67–15.43	9.77–16.66	9.48–18.14
Noncompliance (00079)	12.62 (6.91)	8.48 (1.73)	11.07 (3.68)	15.93 (9.52)	0.034 *	5.72–11.23	9.03–13.11	9.89–21.98
Bathing self-care deficit (00108)	12.54 (4.89)	13.23 (2.53)	15.27 (3.48)	8.91 (4.83)	0.001 *	9.21–17.25	13.34–17.19	5.84–11.98
Sensory perception disturbance: visual, auditory, etc. (00122)	11.77 (3.71)	10.45 (2.28)	12.80 (3.16)	10.91 (4.53)	0.326	6.83–14.07	11.05–14.55	8.03–13.79
Impaired walking (00088)	11.25 (4.46)	11.73 (1.80)	12.59 (4.68)	9.41 (4.39)	0.182	8.87–14.58	9.99–15.18	6.62–12.20
Impaired physical mobility (00085)	10.04 (3.28)	8.40 (1.31)	11.87 (3.27)	8.29 (2.51)	0.006 *	6.31–10.49	10.06–13.68	6.70–9.88
Dressing self-care deficit (00109)	10.03 (4.21)	10.78 (1.62)	12.68 (2.93)	6.48 (3.64)	<0.001 *	8.20–13.36	11.06–14.30	4.16–8.79
Ineffective health maintenance (00099) ^c^	9.10 (8.50)	13.45 (5.43)	11.80 (9.00)	5.95 (5.28)	0.033 *	23.00	18.13	11.00
Impaired home maintenance (00098)	8.00 (4.14)	8.05 (2.63)	9.06 (3.52)	6.65 (5.06)	0.334	3.86–12.24	7.11–11.01	3.43–9.87
Other nursing diagnoses with statistically significant differences								
Constipation (00011)	5.51 (2.28)	5.18 (0.87)	6.69 (1.69)	4.16 (2.53)	0.011 *	3.80–6.55	5.75–7.62	2.55–5.76
Impaired tissue integrity (00044)	5.36 (2.70)	5.55 (1.05)	6.81 (2.77)	3.48 (1.75)	0.003 *	3.88–7.22	5.27–8.34	2.36–4.59
Decreased diversional activity engagement (00097) ^c^	5.10 (4.0)	4.10 (2.45)	6.50 (3.30)	3.70 (2.35)	0.018 *	12.38	20.77	11.25
Feeding self-care deficit (00102)	6.22 (2.83)	5.20 (0.46)	7.91 (2.49)	4.43 (2.46)	0.002 *	4.48–5.92	6.53–9.29	2.86–5.99
Toileting self-care deficit (00110)	6.45 (2.86)	6.58 (1.24)	8.23 (1.66)	4.18 (2.91)	<0.001 *	4.61–8.54	7.31–9.14	2.34–6.03
Risk-prone health behavior (00188)	1.62 (1.15)	0.75 (0.71)	2.18 (1.28)	1.20 (0.71)	0.019 *	0.39–1.89	1.47–2.89	0.75–1.65
Risk for bleeding (00206) ^c^	6.50 (10.30)	6.40 (3.13)	4.40 (3.80)	14.25 (11.40)	0.011 *	15.25	11.40	22.00

^a^ Mean (SD) or Median (IQR): mean (standard deviation) or median (interquartile range). ^b^ 95% CIs: 95% confidence intervals. ^c^ Non-normally distributed variable. * Statistically significant *p*-value.

**Table 4:** Correlations between the prevalence of dysfunctionality determined by Gordon’s health patterns and sociodemographic, financial, and clinical characteristics.

Characteristics	Gordon’s Health Patterns ^a^										
	1	2 ^b^	3	4	5	6	7	8	9	10	11 ^b^
Basic population characteristics											
Population (no. of inhabitants) ^b^					r 0.38 0.035 *						
Age (years)			r 0.43 0.016 *	r 0.40 0.025 *		r 0.46 0.010 *					
Age of HCCPs ^c^ (years)			r 0.50 0.005 *	r 0.43 0.017 *							
Women (%)							r 0.39 0.030 *				
Female HCCPs (%)											
Youth index			r −0.38 0.038 *	r −0.46 0.038 *		r −0.38 0.035 *					
Crude death rate per 100,000 inhabitants				r 0.49 0.006 *							
Non-Spanish nationals (%) ^b^			r −0.46 0.009 *	r −0.37 0.042 *		r −0.43 0.016 *					
Population aged 65 and over (%)				r 0.38 0.034 *							
HCCP population (%)			r 0.45 0.010 *	**r 0.53** 0.002 *		r 0.43 0.017 *					r 0.39 0.028 *
HCCP population aged < 65 years (%)			**r −0.55** 0.001 *	r −0.45 0.011 *							
HCCP population aged 65–79 years (%)											
HCCP population aged ≥ 80 years (%)			**r 0.52** 0.003 *	**r 0.51** 0.004 *		r 0.48 0.007 *					
Level of education											
No education (%)											
Compulsory education (%)											
Secondary education or vocational training (%)											
University education (%)											
Financial characteristics											
Mean gross annual income (euros) ^b^											
Mean disposable annual income (euros) ^b^											
Employed population in the previous four-month period (%) ^b^						r −0.40 0.027 *					
Unemployed population in the previous four-month period (%) ^b^											
Salaried employees (%)			r −0.37 0.042 *								
Self-employed (%)			r 0.37 0.042 *								
Agricultural sector (%) ^b^											
Construction sector (%) ^b^											
Service industries (%) ^b^						r −0.45 0.010 *					r 0.36 0.045 *
Trade/Commerce (%)											
Hospitality sector (%) ^b^		r −0.42 0.020 *		r −0.37 0.043 *							
Social characteristics											
No. of people assisted by Social Services ^b^					r 0.39 0.031 *						
No. of families assisted by Social Services ^b^					r 0.50 0.005 *						
No. of social security benefits granted to the elderly ^b^					r 0.47 0.007 *						
No. of social security benefits granted to persons with a disability/illness ^b^											
Households with 1 person (%)											
Households with ≥5 persons (%)	r ^d^ 0.36 0.044 *	r 0.40 0.024 *		r 0.39 0.031 *							
Clinical characteristics (HCCP population)											
≥65 years, autonomous (%)											
≥65 years, frail (%)											
≥65 years, dependent (%)											
Very high complexity (Pc ≥ 99.5) (%)											
Housebound (%)			r 0.44 0.013 *								
Admitted to a hospital in the previous year (%) ^b^									r −0.37 0.040 *		
Seen by a specialist other than a family doctor in the previous year (%) ^b^											
Good dietary habits (%)											
Regular physical exercise (%)											
Mean no. of visits/year to their family doctor											
Mean no. of visits/year to their primary care nurse	r −0.41 0.023 *			r −0.37 0.040 *	r −0.45 0.011 *					r −0.39 0.032 *	
Prevalence of conditions in HCCPs (%)											
Cardiovascular conditions											
High blood pressure											
Hyperlipidaemia											
Dysrhythmia ^b^											
Cardiac conduction disorder					r −0.39 0.031 *						
Ischaemic heart disease											
Heart failure					r −0.47 0.008 *			r −0.41 0.021 *			r −0.39 0.029 *
Valvular heart disease			r 0.36 0.045 *	r 0.44 0.014 *		r 0.41 0.022 *					
Aortic aneurysm							r −0.38 0.035 *				
Cerebrovascular accident											
Endocrinal-metabolic conditions											
Diabetes mellitus											
Thyroid disorder ^b^						r 0.40 0.025 *	r 0.41 0.023 *				
Obesity ^b^											
Respiratory conditions											
Asthma											
Chronic obstructive pulmonary disease									r 0.47 0.007 *		
Pneumonia											
Musculoskeletal conditions											
Osteoarthritis		r 0.43 0.016 *	r 0.47 0.008 *	r 0.50 0.004 *		r 0.45 0.011 *					
Arthritis			r 0.42 0.020 *	r 0.46 0.009 *	**r 0.52** 0.003 *	r 0.43 0.017 *	r 0.36 0.048 *	r 0.49 0.005 *		r 0.42 0.018 *	
Osteoporosis		r 0.42 0.018 *	r 0.47 0.007 *	r 0.43 0.017 *		r 0.37 0.042 *	r 0.43 0.016 *				
Nervous system/neurodegenerative conditions											
Dementia ^b^											
Parkinson’s ^b^											
Epilepsy											
Paralysis											
Mental health conditions											
Depression											
Anxiety									r −0.37 0.041 *		
Schizophrenia	r 0.38 0.034 *			r 0.40 0.027 *						r 0.36 0.045 *	
Alcohol use disorder											
Substance abuse ^b^											
Suicidal behaviour or suicide attempt											
Liver/kidney conditions											
Chronic renal failure											
Liver disease ^b^	r 0.44 0.013 *	r 0.50 0.004 *			r 0.42 0.018 *						r 0.36 0.046 *
Oncological conditions											
Prior neoplasm											
Active neoplasm ^b^											
Metastasis											
Non-Hodgkin lymphoma					r −0.47 0.008 *			r −0.40 0.025 *			
Other											
Urinary tract infection											
Septicaemia ^b^											
Lupus						r 0.40 0.024 *					
Glaucoma											
Anaemia											
Pharmacological treatment in HCCPs (%)											
Analgesics	r 0.49 0.005 *		**r 0.53** 0.002 *		r 0.37 0.040 *						
Antidepressants											
Anxiolytics											
Opioids					r 0.49 0.005 *						
Hypnotics/sedatives		r −0.36 0.045 *									
Antipsychotics											
Anti-dementia drugs											

^a^ 1: Health perception/Health management; 2: Nutrition/Metabolism; 3: Elimination; 4: Physical activity/Exercise; 5: Sleep/Rest; 6: Cognition/Perception; 7: Self-perception/Self-concept; 8: Role/Relationships; 9: Sexuality/Reproduction; 10: Coping/Stress tolerance; 11: Values/Beliefs. ^b^ Non-normally distributed variable. ^c^ HCCPs: highly complex chronic patients. ^d^ r: Pearson’s or Spearman’s correlation coefficient, as appropriate. * Statistically significant *p*-value. In bold, correlation coefficient values greater than or equal to 0.51 (moderate/strong association).

## Data Availability

The data presented in this study are available upon request from the corresponding author. The data are not publicly available due to privacy/ethical restrictions.

## Supplementary Materials

The following supporting information can be downloaded at https://www.mdpi.com/article/10.3390/nursrep14020096/s1. Table S1. Correlations between prevalence of NANDA-I nursing diagnoses (NDs) and sociodemographic, financial, and clinical characteristics; Figure S1. Municipalities in the healthcare area distributed in metropolitan, northern, and southern areas; Figures S2–S12. Geo-referencing of the prevalence of dysfunctionality in the population, distributed by municipality in the healthcare area under study; Figures S13–S37. Geo-referencing of the most prevalent care needs identified using nursing diagnoses, distributed by municipality in the healthcare area under study.

## Public Involvement Statement

There is no public involvement in any aspect of this research.

## Guidelines and Standards Statement

This manuscript was drafted against “The Strengthening the Reporting of Observational Studies in Epidemiology (STROBE) Statement: guidelines for reporting observational studies”.
