# Supplementary material for: Community Care Needs of Highly Complex Chronic Patients: An Epidemiological Study in a Healthcare Area
Source: Nurs Rep. 2024 May 20;14(2):1260–86. doi: 10.3390/nursrep14020096 (PMC11130826; doi:10.3390/nursrep14020096)

Supplementary materials

Table S1. Correlations between prevalence of NANDA-I nursing diagnoses (NDs) and sociodemographic, financial, and clinical characteristics.
Figure S1. Municipalities in the healthcare area distributed in metropolitan, northern, and southern areas.
Figure S2-Figure S12. Geo-referencing of the prevalence of dysfunctionality in the population, distributed by municipality in the healthcare area under study.
Figure S13-Figure S37. Geo-referencing of the most prevalent care needs identified using nursing diagnoses, distributed by municipality in the health area under study.

Table S1. Correlations between the prevalence of NANDA-I nursing diagnoses and socio-demographic, financial, and clinical characteristics (I).

Characteristics	Mean no. of NDs	Nursing diagnoses by NANDA-I code [a]														
		00162[b]	00186	00132	00046	00214	00032	00155	00043[b]	00016	00179[b]	00001	00035[b]	00004[b]	00084	00078[b]
Basic population characteristics																
Population (no. of inhabitants)[b]						**r 0.58**<br>**0.001***					**r -0.65**<br>**<0.001***				r 0.38<br>0.034*	
Age (years)	r^{d} 0.44<br>0.013*					**r -0.67**<br>**<0.001***		**r 0.72**<br>**<0.001***	r 0.38<br>0.035*		***r 0.80***<br>***<0.001****					
Age of HCCPs[c] (years)	r 0.40<br>0.027*					**r -0.67**<br>**<0.001***		**r 0.72**<br>**<0.001***			**r 0.59**<br>**<0.001***					
Women (%)					r 0.40<br>0.024*											
Female HCCPs (%)																
Youth Index						**r 0.59**<br>**<0.001***		**r -0.56**<br>**0.001***			**r -0.69**<br>**<0.001***					
Crude death rate per 100,000 inhabitants	r 0.45<br>0.011*					r -0.36<br>0.047*		**r 0.62**<br>**<0.001***			**r 0.60**<br>**<0.001***					
Non-Spanish nationals (%)[b]									r -0.49<br>0.005*					r -0.49<br>0.005*		
Population aged 65 and over (%)	r 0.49<br>0.005*					**r -0.61**<br>**<0.001***		**r 0.71**<br>**<0.001***			**r 0.75**<br>**<0.001***			r 0.36<br>0.050*		
HCCP population (%)	r 0.47<br>0.007*					r -0.45<br>0.011*		**r 0.66**<br>**<0.001***	r 0.46<br>0.010*		r 0.48<br>0.007*			r 0.50<br>0.004*		
HCCP population <65 years (%)	r -0.41<br>0.023*					**r 0.65**<br>**<0.001***		**r -0.62**<br>**<0.001***	r -0.42<br>0.019*	r -0.37<br>0.041*	**r -0.61**<br>**<0.001***			r -0.50<br>0.004*		
HCCP population 65-79 years (%)																
HCCP population ≥80 years (%)						**r -0.69**<br>**<0.001***		**r 0.70**<br>**<0.001***			**r 0.62**<br>**<0.001***					
Level of education																
No education (%)	r 0.49<br>0.005*		r 0.49<br>0.005*	**r 0.51**<br>**0.004***	r 0.49<br>0.006*		r 0.48<br>0.006*					r 0.41<br>0.021*	r 0.46<br>0.009*			
Compulsory education (%)											r 0.38<br>0.038*					
Secondary education or vocational training (%)		r -0.44<br>0.014*						r -0.38<br>0.037*	r -0.38<br>0.035*							
University education (%)																
Financial characteristics																
Mean gross annual income (euros)[b]		**r -0.60**<br>**<0.001***														

Mean disposable annual income (euros)[b]	**r -0.61 <0.001***									
Population in work in the previous four-month period (%)[b]				r 0.38 0.035*		r -0.38 0.036*				
Unemployed population in the previous four-month period (%)[b]						r 0.37 0.039*				
Salaried employees (%)						r -0.48 0.006*			**r -0.51 0.003***	
Self-employed (%)						r 0.48 0.006*			**r 0.51 0.003***	
Agricultural sector (%)[b]	r 0.39 <0.029*						**r 0.52 0.003***			
Construction sector (%)[b]								r 0.38 0.037*		
Service industries (%)[b]							r -0.43 0.017*			
Trade/Commerce (%)										
Hospitality sector (%)[b]										
Social characteristics										
No. of people assisted by Social Services [b]		r 0.41 0.021*	r 0.36 0.044*	**r 0.55 0.001***			r -0.39 0.032*			
No. of families assisted by Social Services[b]				r 0.37 0.045*						
No. of social security benefits granted to the elderly[b]										
No. of social security benefits granted to persons with a disability/illness[b]										
Households with 1 person (%)								r -0.45 0.012*		
Households with ≥5 persons (%)						r 0.36 0.044*				
Clinical characteristics (HCCP population)										
≥65 years, autonomous (%)					r -0.39 0.030*					
≥65 years, frail (%)										
≥65 years, dependent (%)							r 0.40 0.025*			
Very high complexity (Pc≥99.5) (%)										r 0.36 0.048*

Housebound (%)						**r -0.62 <0.001***		r 0.50 0.005*								
Admitted to hospital in the previous year (%)[b]									***r -0.78 <0.001****	r -0.42 0.019*				r -0.45 0.012*		
Seen by a specialist other than a family doctor in the previous year (%)[b]		r -0.39 0.031*		r -0.37 0.042*	r -0.39 0.032*		**r -0.52 0.003***			r -0.38 0.034*						
Good dietary habits (%)																
Regular physical exercise (%)											r -0.42 0.021*					r -0.44 0.016*
Mean no. of visits/year to their family doctor						r -0.38 0.033*									r -0.41 0.021*	
Mean no. of visits/year to their primary care nurse		**r 0.55 0.001***							r 0.43 0.016*						r -0.39 0.030*	
Prevalence of conditions among HCCPs (%)																
Cardiovascular conditions																
High blood pressure																
Hyperlipidaemia	r 0.47 0.008*	r 0.39 <0.032*	r 0.49 0.005*	r 0.42 0.018*	r 0.39 0.028*				r 0.38 0.035*	**r 0.65 <0.001***			r 0.41 0.021*			
Dysrhythmia[b]																
Cardiac conduction disorder																
Ischaemic heart disease	r -0.40 0.026*					r 0.38 0.036*		r -0.48 0.006*	**r -0.60 <0.001***	r -0.49 0.005*				r -0.47 0.008*		
Heart failure																
Valvular heart disease									**r 0.63 <0.001***	r 0.47 0.008*						
Aortic aneurysm																
Cerebrovascular accident		r -0.36 0.049*														
Endocrinal-metabolic conditions																
Diabetes mellitus									r 0.44 0.012*	r 0.37 0.038*						
Thyroid disorder[b]																
Obesity[b]							r 0.38 0.035*		r 0.46 0.009*	r 0.40 0.025*						
Respiratory conditions																
Asthma	r 0.41 0.024*							r 0.44 0.014*			r 0.36 0.048*			r 0.39 0.031*		
Chronic obstructive pulmonary disease									r -0.41 0.022*							
Pneumonia																
Musculoskeletal conditions																

Osteoarthritis	r 0.45<br>0.011*					r -0.36<br>0.045*		**r 0.57**<br>**0.001***	r 0.43<br>0.017*					r 0.38<br>0.035*		
Arthritis																
Osteoporosis																
Nervous system/neurodegenerative conditions																
Dementia[b]																
Parkinson's[b]																
Epilepsy																
Paralysis					r 0.40<br>0.028*				r 0.37<br>0.044*						r 0.38<br>0.038*	
Mental health conditions																
Depression			r 0.36<br>0.050*		r 0.43<br>0.017*										r 0.36<br>0.046*	
Anxiety	r 0.37<br>0.042*		**r 0.56**<br>**0.001***	r 0.47<br>0.008*	r 0.47<br>0.007*									r 0.41<br>0.021*	r 0.43<br>0.015*	
Schizophrenia	r 0.42<br>0.020*					r -0.50<br>0.004*		r 0.50<br>0.004*		r 0.39<br>0.032*	r 0.45<br>0.012*					r 0.37<br>0.041*
Alcohol use disorder																
Substance abuse[b]																
Suicidal behaviour or suicide attempt																
Liver/kidney conditions																
Chronic renal failure	r -0.38<br>0.035*		**r -0.52**<br>**0.003***	**r -0.56**<br>**0.001***	**r -0.56**<br>**0.001***		**r -0.51**<br>**0.004***		r -0.44<br>0.013*	r -0.38<br>0.036*						
Liver disease[b]																
Oncological conditions																
Prior neoplasm																
Active neoplasm[b]																
Metastasis											r -0.43<br>0.017*					
Non-Hodgkin lymphoma																r -0.47<br>0.007*
Other																
Urinary tract infection	**r 0.51**<br>**0.003***				r 0.40<br>0.027*	r -0.39<br>0.030*		**r 0.63**<br>**<0.001***	r 0.41<br>0.023*	r 0.47<br>0.008*	r 0.44<br>0.013*					
Septicaemia[b]									**r 0.60**<br>**<0.001***							
Lupus					r 0.37<br>0.041*								r 0.42<br>0.018*			
Glaucoma						r 0.39<br>0.031*									r 0.42<br>0.017*	

Anaemia								r 0.39<br>0.029*	r 0.45<br>0.012*					r 0.41<br>0.024*	
Pharmacological treatments among HCCPs (%)															
Analgesics						r -0.46<br>0.010*									r -0.43<br>0.017*
Antidepressants	r 0.50<br>0.005*				r 0.38<br>0.035*	r -0.41<br>0.021*		**r 0.51**<br>**0.004***		**r 0.52**<br>**0.003***				r 0.41<br>0.022*	
Anxiolytics						r -0.36<br>0.044*								r 0.41<br>0.023*	
Opioids													r 0.46<br>0.009*		
Hypnotics/sedatives															
Antipsychotics	r 0.36<br>0.047*							r 0.48<br>0.006*	r 0.43<br>0.015*						
Anti-dementia drugs								r 0.46<br>0.009*							

[a] Nursing diagnoses by NANDA-I code: Readiness for enhanced health management [00162]; Willingness to improve immunization status [00186]; Acute pain [00132]; Impaired skin integrity [00046]; Impaired comfort [00214]; Ineffective breathing pattern [00032]; Risk for falls [00155]; Ineffective protection [00043]; Impaired urinary elimination [00016]; Risk for unstable blood glucose level [00179]; Nutritional imbalance: excess [00001]; Risk for injury [00035]; Risk for infection [00004]; Health-generating behaviors [00084]; Ineffective health management [00078]. [b] Non-normally distributed variable. [c] HCCPS: highly complex chronic patients. [d] r: Pearson's or Spearman's correlation coefficient, as appropriate. * Statistically significant p-value. In bold, correlation coefficient values between 0.51-0.75 (moderate/strong association). In bold and italics, correlation coefficient values between 0.76-1.00 (strong/perfect association).

Table S1. Correlations between the prevalence of NANDA-I nursing diagnoses and socio-demographic, financial, and clinical characteristics (II).

Characteristics	Nursing diagnoses by NANDA-I code [a]														
	00133	00146	00079	00108	00122	00088	00099[b]	00109	00085	00182	00002	00011	00020	00040	00044
Basic population characteristics															
Population (no. of inhabitants)[b]	**r[d] -0.55<br>0.001***		r 0.46<br>0.010*							r 0.47<br>0.007*	**r -0.52<br>0.003***	r -0.44<br>0.013*	r -0.42<br>0.020*		
Age (years)	**r 0.67<br><0.001***		r -0.46<br>0.009*	**r 0.67<br><0.001***		**r 0.68<br><0.001***		**r 0.66<br><0.001***			**r 0.67<br><0.001***	**r 0.64<br><0.001***	**r 0.59<br><0.001***		
Age of HCCPs[c] (years)	**r 0.57<br>0.001***		r -0.41<br>0.023*	**r 0.72<br><0.001***		**r 0.58<br>0.001***	r 0.39<br>0.029*	**r 0.72<br><0.001***	r 0.47<br>0.008*		**r 0.64<br><0.001***	**r 0.63<br><0.001***	**r 0.75<br><0.001***		
Women (%)															
Female HCCPs (%)															
Youth Index	**r -0.56<br>0.001***	r -0.41<br>0.020*	r 0.36<br>0.050*	**r -0.52<br>0.003***		**r -0.63<br><0.001***		**r -0.51<br>0.003***			**r -0.52<br>0.003***	r -0.49<br>0.005*	r -0.43<br>0.015*	r -0.40<br>0.025*	
Crude death rate per 100,000 inhabitants	r 0.44<br>0.012*			**r 0.64<br><0.001***		**r 0.67<br><0.001***		**r 0.60<br><0.001***			**r 0.51<br>0.003***	**r 0.52<br>0.003***	**r 0.53<br>0.002***		
Non-Spanish nationals (%)[b]				**r -0.53<br>0.002***			**r -0.51<br>0.003***	**r -0.57<br>0.001***	r -0.41<br>0.021*		r -0.36<br>0.047*	r -0.39<br>0.028*	r -0.40<br>0.028*		r -0.42<br>0.020*
Population aged 65 and over (%)	**r 0.62<br><0.001***		r -0.36<br>0.044*	**r 0.69<br><0.001***		**r 0.67<br><0.001***	r 0.38<br>0.036*	**r 0.67<br><0.001***	r 0.41<br>0.023*		**r 0.67<br><0.001***	**r 0.60<br><0.001***	**r 0.58<br>0.001***		
HCCP population (%)	**r 0.51<br>0.003***		r -0.40<br>0.025*	**r 0.73<br><0.001***		**r 0.66<br><0.001***		**r 0.75<br><0.001***	**r 0.58<br>0.001***		**r 0.54<br>0.002***	**r 0.55<br>0.001***	**r 0.59<br>0.001***		
HCCP population <65 years (%)	**r -0.58<br>0.001***		r 0.40<br>0.024*	**r -0.64<br><0.001***		r -0.48<br>0.006*	r -0.40<br>0.026*	**r -0.66<br><0.001***	**r -0.53<br>0.002***		**r -0.62<br><0.001***	**r -0.61<br><0.001***	**r -0.69<br><0.001***		
HCCP population 65-79 years (%)															
HCCP population ≥80 years (%)	**r 0.53<br>0.002***		**r -0.53<br>0.002***	**r 0.63<br><0.001***		**r 0.53<br>0.002***		**r 0.62<br><0.001***	r 0.39<br>0.032*		**r 0.62<br><0.001***	**r 0.55<br>0.001***	**r 0.68<br><0.001***		
Level of education															
No education (%)		r 0.36<br>0.044*													
Compulsory education (%)															
Secondary education or vocational training (%)						r -0.37<br>0.039*									
University education (%)				r 0.41<br>0.021*		r 0.44<br>0.014*	r 0.39<br>0.030*								
Financial characteristics															
Mean gross annual income (euros)[b]	**r -0.57<br>0.001***	r -0.37<br>0.042*													

Mean disposable annual income (euros)[b]	**r -0.57**<br>**0.001***	r -0.35<br>0.050*													
Population in work in the previous four-month period (%)[b]	r -0.43<br>0.015*			r -0.43<br>0.017*				r -0.44<br>0.014*	r -0.44<br>0.013*			r -0.47<br>0.008*	r -0.47<br>0.007*		
Unemployed population in the previous four-month period (%)[b]															
Salaried employees (%)				r -0.36<br>0.046*				r -0.42<br>0.019*	r -0.41<br>0.021*			r -0.43<br>0.016*	r -0.37<br>0.039*		
Self-employed (%)				r 0.36<br>0.046*				r -0.42<br>0.019*	r 0.41<br>0.021*			r 0.43<br>0.016*	r 0.37<br>0.039*		
Agricultural sector (%)[b]	**r 0.57**<br>**0.001***			r 0.37<br>0.044*									r 0.40<br>0.027*		
Construction sector (%)[b]															
Service industries (%)[b]			r 0.40<br>0.028*												
Trade/Commerce (%)															
Hospitality sector (%)[b]			r 0.41<br>0.024*				r -0.49<br>0.005*								
Social characteristics															
No. of people assisted by Social Services [b]										r 0.43<br>0.016*					r 0.48<br>0.007*
No. of families assisted by Social Services[b]										r 0.49<br>0.006*					
No. of social security benefits granted to the elderly[b]															r 0.42<br>0.018*
No. of social security benefits granted to persons with a disability/illness[b]										r 0.45<br>0.011*					r 0.44<br>0.013*
Households with 1 person (%)															
Households with ≥5 persons (%)	r -0.39<br>0.029*														**r 0.56**<br>**0.001***
Clinical characteristics (HCCP population)															
≥65 years, autonomous (%)				r -0.39<br>0.032*									r -0.38<br>0.033*		
≥65 years, frail (%)															
≥65 years, dependent (%)			r -0.43<br>0.017*								r 0.43<br>0.015*		**r 0.52**<br>**0.002***		
Very high complexity (Pc≥99.5) (%)			r -0.40<br>0.028*												
Housebound (%)			r -0.47<br>0.008*	r 0.36<br>0.049*			r 0.42<br>0.020*	r 0.40<br>0.026*			r 0.48<br>0.006*		**r 0.59**<br>**0.001***		

Admitted to hospital in the previous year (%)[b]				**r -0.57 0.001***		r -0.38 0.038*		**r -0.67 <0.001***	r -0.40 0.026*			r -0.40 0.027*			r -0.49 0.005*
Seen by a specialist other than a family doctor in the previous year (%)[b]															
Good dietary habits (%)						r 0.38 0.038*									
Regular physical exercise (%)	r -0.39 0.033*	**r -0.59 0.001***			r -0.38 0.041*										
Mean no. of visits/year to their family doctor	r 0.57 0.001*	r 0.48 0.007*													r -0.37 0.039*
Mean no. of visits/year to their primary care nurse															
Prevalence of conditions among HCCPs (%)															
Cardiovascular conditions															
High blood pressure									r 0.38 0.033*						
Hyperlipidaemia									r 0.46 0.009*						r 0.41 0.023*
Dysrhythmia[b]								r 0.36 0.049*		r -0.46 0.010*	r 0.39 0.030*				
Cardiac conduction disorder															
Ischaemic heart disease				r -0.50 0.004*		r -0.45 0.012*		r -0.50 0.005*				r -0.40 0.026*			
Heart failure															
Valvular heart disease			r -0.39 0.031*					r 0.37 0.039*	r 0.42 0.020*						r 0.46 0.010*
Aortic aneurysm															
Cerebrovascular accident															
Endocrinal-metabolic conditions															
Diabetes mellitus														r -0.38 0.036*	r 0.36 0.049*
Thyroid disorder[b]												r 0.43 0.017*			
Obesity[b]												r 0.37 0.042*			r 0.47 0.008*
Respiratory conditions															
Asthma				r 0.39 0.032*		r 0.47 0.007*	r 0.41 0.021*					r 0.40 0.020*			
Chronic obstructive pulmonary disease				r -0.40 0.027*				r -0.40 0.025*							
Pneumonia															

Musculoskeletal conditions															
Osteoarthritis			r -0.37<br>0.041*	**r 0.57<br>0.001***		**r 0.54<br>0.002***	r 0.40<br>0.027*	**r 0.65<br><0.001***	r 0.46<br>0.010*			r 0.49<br>0.006*	r 0.46<br>0.009*	**r 0.51<br>0.003***	r 0.45<br>0.010*
Arthritis														r 0.48<br>0.006*	
Osteoporosis								r 0.38<br>0.034*	r 0.36<br>0.046*						
Nervous system/neurodegenerative conditions															
Dementia[b]															
Parkinson’s[b]			r 0.37<br>0.038*				r -0.45<br>0.012*								
Epilepsy		**r -0.51<br>0.004***													
Paralysis									r 0.44<br>0.014*						r 0.41<br>0.021*
Mental health conditions															
Depression															
Anxiety															r 0.39<br>0.029*
Schizophrenia	r 0.46<br>0.009*	**r 0.55<br>0.001***			r 0.43<br>0.017*			r 0.39<br>0.033*			r 0.43<br>0.015*	r 0.49<br>0.005*			
Alcohol use disorder															
Substance abuse[b]															
Suicidal behaviour or suicide attempt															
Liver/kidney conditions															
Chronic renal failure				r -0.37<br>0.042*		r -0.36<br>0.046*	r -0.37<br>0.043*			r -0.37<br>0.042*					r -0.46<br>0.009*
Liver disease[b]															
Oncological conditions															
Prior neoplasm															
Active neoplasm[b]															
Metastasis	r -0.38<br>0.035*	**r -0.53<br>0.002***													
Non-Hodgkin lymphoma		r -0.36<br>0.048*		r 0.39<br>0.032*				r 0.41<br>0.021*							
Other															
Urinary tract infection	r 0.43<br>0.017*			**r 0.67<br><0.001***		**r 0.54<br>0.002***		**r 0.66<br><0.001***	**r 0.55<br>0.001***		r 0.44<br>0.014*	r 0.46<br>0.009*	**r 0.62<br><0.001***		r 0.42<br>0.018*
Septicaemia[b]				r 0.43<br>0.016*				r 0.49<br>0.006*							**r 0.52<br>0.003***

Lupus															
Glaucoma	r -0.43 0.017*														
Anaemia				r 0.50 0.004*		r 0.37 0.041*		r 0.48 0.007*					r 0.41 0.022*		r 0.37 0.041*
Pharmacological treatments among HCCPs (%)															
Analgesics	**r 0.52 0.003***	**r 0.59 <0.001***								r -0.36 0.046*	r 0.44 0.014*			r 0.38 0.037*	
Antidepressants		r 0.39 0.032*					r 0.44 0.012*	r 0.45 0.011*	r 0.40 0.027*		**r 0.52 0.003***	r 0.38 0.033*	r 0.45 0.012*		r 0.41 0.022*
Anxiolytics		r 0.43 0.016*									r 0.41 0.023*				
Opioids															
Hypnotics/sedatives										r 0.39 0.029*					
Antipsychotics				r 0.50 0.004*				**r 0.53 0.002***			r 0.48 0.006*	r 0.39 0.028*	r 0.42 0.020*		r 0.37 0.038*
Anti-dementia drugs								r 0.40 0.027*							

[a] Nursing diagnoses by NANDA-I code: Chronic pain [00133]; Anxiety [00146]; Noncompliance [00079]; Bathing self-care deficit [00108]; Sensory perception disturbance: visual, auditory, kinaesthetic, tactile [00122]; Impaired walking [00088]; Ineffective health maintenance [00099]; Dressing self-care deficit [00109]; Impaired physical mobility [00085]; Readiness for enhanced self-care [00182]; Imbalanced nutrition: less than body requirements [00002]; Constipation [00011]; Functional urinary incontinence [00020]; Risk for disuse syndrome [00040]; Impaired tissue integrity [00044]. [b] Non-normally distributed variable. [c] HCCPS: highly complex chronic patients. [d] r: Pearson's or Spearman's correlation coefficient, as appropriate. * Statistically significant p-value. In bold, correlation coefficient values between 0.51-0.75 (moderate/strong association). In bold and italics, correlation coefficient values between 0.76-1.00 (strong/perfect association).

Table S1. Correlations between the prevalence of NANDA-I nursing diagnoses and socio-demographic, financial, and clinical characteristics (III).

Characteristics	Nursing diagnoses by NANDA-I code [a]														
	**00047[b]**	**00051**	**00052[b]**	**00053[b]**	**00054**	**00061**	**00062[b]**	**00069[b]**	**00092**	**00094**	**00095**	**00097[b]**	**00098**	**00101**	**00102**
Basic population characteristics															
Population (no. of inhabitants)[b]															
Age (years)	r[d] 0.40<br>0.028*	r 0.40<br>0.025*			**r 0.55**<br>**0.002***	r 0.36<br>0.045*			r 0.47<br>0.008*	r 0.42<br>0.019*	r 0.46<br>0.009*	r 0.37<br>0.040*	**r 0.72**<br>**<0.001***	**r 0.60**<br>**<0.001***	**r 0.58**<br>**0.001***
Age of HCCPs[c] (years)	r 0.37<br>0.043*	r 0.36<br>0.046*	r 0.36<br>0.048*		r 0.41<br>0.020*	r 0.45<br>0.011*					r 0.43<br>0.016*	r 0.41<br>0.022*	**r 0.65**<br>**<0.001***	r 0.42<br>0.018*	**r 0.52**<br>**0.003***
Women (%)															
Female HCCPs (%)									r 0.40<br>0.028*						
Youth Index	r -0.45<br>0.011*	r -0.38<br>0.035*			r -0.40<br>0.027*				r -0.43<br>0.017*		r -0.36<br>0.045*	r -0.44<br>0.014*	**r 0.62**<br>**<0.001***	r -0.44<br>0.014*	r -0.45<br>0.011*
Crude death rate per 100,000 inhabitants			r 0.49<br>0.006*	r 0.49<br>0.005*	**r 0.58**<br>**0.001***					r 0.36<br>0.048*	r 0.36<br>0.048*	r 0.41<br>0.022*	**r 0.67**<br>**<0.001***	r 0.45<br>0.011*	**r 0.55**<br>**0.001***
Non-Spanish nationals (%)[b]		r -0.43<br>0.016*											r -0.39<br>0.029*		r -0.39<br>0.032*
Population aged 65 and over (%)	**r 0.53**<br>**0.002***	r 0.47<br>0.008*	r 0.42<br>0.018*		**r 0.61**<br>**<0.001***				**r 0.51**<br>**0.003***	r 0.40<br>0.025*	r 0.40<br>0.026*	**r 0.55**<br>**0.001***	**r 0.68**<br>**<0.001***	**r 0.53**<br>**0.002***	**r 0.63**<br>**<0.001***
HCCP population (%)	r 0.49<br>0.005*	r 0.41<br>0.023*	**r 0.55**<br>**0.001***	**r 0.54**<br>**0.002***	r 0.48<br>0.006*	r 0.45<br>0.012*			r 0.37<br>0.040*		r 0.39<br>0.032*	**r 0.54**<br>**0.002***	**r 0.64**<br>**<0.001***	r 0.48<br>0.007*	**r 0.68**<br>**<0.001***
HCCP population <65 years (%)	r -0.41<br>0.022*					r -0.41<br>0.021*					r -0.48<br>0.006*	r -0.47<br>0.008*	**r -0.56**<br>**0.001***		r -0.47<br>0.007*
HCCP population 65-79 years (%)													r -0.39<br>0.030*	r -0.41<br>0.024*	
HCCP population ≥80 years (%)					r 0.41<br>0.022*	r 0.39<br>0.030*					r 0.42<br>0.019*		r 0.69<br><0.001*	r 0.50<br>0.004*	r 0.44<br>0.014*
Level of education															
No education (%)				r 0.42<br>0.019*				r 0.43<br>0.017*				r 0.36<br>0.048*			
Compulsory education (%)															
Secondary education or vocational training (%)		r -0.37<br>0.039*	r -0.36<br>0.045*	r -0.40<br>0.026*											
University education (%)									r 0.46<br>0.010*			r 0.37<br>0.041*			r 0.44<br>0.015*
Financial characteristics															
Mean gross annual income (euros)[b]															

Mean disposable annual income (euros)[b]														
Population in work in the previous four-month period (%)[b]					r -0.37<br>0.042*									r -0.36<br>0.049*
Unemployed population in the previous four-month period (%)[b]									r -0.50<br>0.005*					
Salaried employees (%)										r -0.39<br>0.029*	r -0.36<br>0.050*			r -0.38<br>0.033*
Self-employed (%)										r 0.39<br>0.029*	r 0.36<br>0.050*			r 0.38<br>0.033*
Agricultural sector (%)[b]														
Construction sector (%)[b]														
Service industries (%)[b]														
Trade/Commerce (%)						r 0.37<br>0.041*								
Hospitality sector (%)[b]									r -0.38<br>0.037*				r -0.44<br>0.013*	
Social characteristics														
No. of people assisted by Social Services [b]														
No. of families assisted by Social Services[b]														
No. of social security benefits granted to the elderly[b]														
No. of social security benefits granted to persons with a disability/illness[b]														
Households with 1 person (%)														
Households with ≥5 persons (%)														
Clinical characteristics (HCCP population)														
≥65 years, autonomous (%)												r -0.36<br>0.047*	r -0.40<br>0.025*	r -0.40<br>0.026*
≥65 years, frail (%)														
≥65 years, dependent (%)						r 0.40<br>0.028*						r 0.40<br>0.027*	r 0.44<br>0.013*	
Very high complexity (Pc≥99.5) (%)						r -0.48<br>0.007*								
Housebound (%)										r 0.43<br>0.016*		r 0.44<br>0.013*		
Admitted to hospital in the previous year (%)[b]	r -0.37<br>0.040*													r -0.46<br>0.010*

Seen by a specialist other than a family doctor in the previous year (%)[b]							**r 0.63<br><0.001***								
Good dietary habits (%)					r 0.38<br>0.033*					r 0.41<br>0.023*				r 0.36<br>0.044*	r 0.36<br>0.044*
Regular physical exercise (%)		r -0.38<br>0.036*	r -0.39<br>0.036*					r -0.46<br>0.010*			r -0.38<br>0.039*				
Mean no. of visits/year to their family doctor							r 0.46<br>0.009*								
Mean no. of visits/year to their primary care nurse															
Prevalence of conditions among HCCPs (%)															
Cardiovascular conditions															
High blood pressure															
Hyperlipidaemia								r 0.36<br>0.045*							
Dysrhythmia[b]															
Cardiac conduction disorder															
Ischaemic heart disease	**r -0.53<br>0.002***	r -0.47<br>0.007*	r -0.41<br>0.022*									r -0.40<br>0.024*	r -0.39<br>0.033*		r -0.41<br>0.022*
Heart failure															
Valvular heart disease															
Aortic aneurysm															
Cerebrovascular accident															
Endocrinal-metabolic conditions															
Diabetes mellitus															
Thyroid disorder[b]			r 0.45<br>0.010*	**r 0.55<br>0.001***								r 0.43<br>0.015*			
Obesity[b]				r 0.38<br>0.036*			r -0.39<br>0.031*								
Respiratory conditions															
Asthma					**r 0.60<br><0.001***					**r 0.52<br>0.003***			r 0.42<br>0.018*	r 0.40<br>0.026*	r 0.48<br>0.006*
Chronic obstructive pulmonary disease													r -0.44<br>0.014*	r -0.41<br>0.021*	r -0.37<br>0.038*
Pneumonia															
Musculoskeletal conditions															
Osteoarthritis	r 0.46<br>0.010*	r 0.45<br>0.010*	r 0.36<br>0.045*	r 0.43<br>0.016*	r 0.37<br>0.042*				r 0.36<br>0.046*	r 0.47<br>0.008*	r 0.48<br>0.006*	r 0.41<br>0.024*	r 0.46<br>0.009*	r 0.41<br>0.022*	r 0.41<br>0.022*
Arthritis															
Osteoporosis											r 0.36				

											0.044*				
Nervous system/neurodegenerative conditions															
Dementia[b]			r 0.47 0.008*	r 0.37 0.040*	r 0.46 0.010*					r 0.44 0.014*					
Parkinson's[b]															
Epilepsy															
Paralysis															
Mental health conditions															
Depression															
Anxiety					r 0.49 0.006*				r 0.36 0.048*			r 0.36 0.048*		r 0.37 0.043*	r 0.49 0.005*
Schizophrenia	r 0.36 0.046*			r 0.38 0.038*				r 0.48 0.006*			**r 0.56 0.001***	r 0.45 0.012*	r 0.46 0.010*	r 0.39 0.032*	
Alcohol use disorder				r 0.38 0.035*											
Substance abuse[b]															
Suicidal behaviour or suicide attempt															
Liver/kidney conditions															
Chronic renal failure		r -0.43 0.015*			r -0.38 0.036*			r 0.37 0.042*							r -0.40 0.025*
Liver disease[b]															
Oncological conditions															
Prior neoplasm								r -0.37 0.038*							
Active neoplasm[b]															
Metastasis							r -0.49 0.006*	r -0.52 0.003*			r -0.48 0.006*				
Non-Hodgkin lymphoma															r 0.38 0.033*
Other															
Urinary tract infection	r 0.47 0.007*	**r 0.56 0.001***			r 0.50 0.004*				r 0.36 0.050*			r 0.38 0.034*	**r 0.54 0.002***	r 0.43 0.016*	**r 0.59 0.001***
Septicaemia[b]															r 0.38 0.038*
Lupus		r 0.39 0.030*					r 0.40 0.027*								
Glaucoma															
Anaemia			r 0.36 0.049*	r 0.36 0.047*									r 0.47 0.008*		r 0.46 0.009*
Pharmacological treatments among HCCPs (%)															

Analgesics	r 0.37<br>0.040*										**r 0.57**<br>**0.001***				
Antidepressants	r 0.36<br>0.048*	r 0.42<br>0.018*		r 0.36<br>0.045*	r 0.44<br>0.014*						**r 0.55**<br>**0.001***	r 0.38<br>0.033*	r 0.38<br>0.033*		
Anxiolytics											r 0.42<br>0.018*				r 0.40<br>0.027*
Opioids															
Hypnotics/sedatives															
Antipsychotics			r 0.45<br>0.011*	r 0.44<br>0.014*	r 0.47<br>0.008*							**r 0.54**<br>**0.002***	r 0.46<br>0.010*		r 0.48<br>0.007*
Anti-dementia drugs	r 0.39<br>0.030*		r 0.44<br>0.013*										r 0.44<br>0.013*		

[a] Nursing diagnoses by NANDA-I code: Risk for impaired skin integrity [00047]; Impaired verbal communication [00051]; Impaired social interaction [00052]; Social interaction [00053]; Risk for loneliness [00054]; Caregiver role strain [00061]; Risk for caregiver role strain [00062]; Ineffective coping [00069]; Activity intolerance [00092]; Risk for activity intolerance [00094]; Insomnia [00095]; Decreased diversional activity engagement [00097]; Impaired home maintenance [00098]; Adult failure to thrive [00101]; Feeding self-care deficit [00102]. [b] Non-normally distributed variable. [c] HCCPS: highly complex chronic patients. [d] r: Pearson's or Spearman's correlation coefficient, as appropriate. * Statistically significant p-value. In bold, correlation coefficient values between 0.51-0.75 (moderate/strong association). In bold and italics, correlation coefficient values between 0.76-1.00 (strong/perfect association).

Table S1. Correlations between the prevalence of NANDA-I nursing diagnoses and socio-demographic, financial, and clinical characteristics (IV).

Characteristics	Nursing diagnoses by NANDA-I code [a]														
	**00110**	**00119**	**00120**	**00124**	**00125[b]**	**00129**	**00130[b]**	**00131**	**00135[b]**	**00136[b]**	**00137[b]**	**00148[b]**	**00153**	**00168[b]**	**00173**
Basic population characteristics															
Population (no. of inhabitants)[b]	r[d] -0.36<br>0.050*										r -0.38<br>0.037*				
Age (years)	**r 0.63<br><0.001***	r 0.43<br>0.015*				**r 0.55<br>0.001***		r 0.49<br>0.005*			r 0.39<br>0.031*		r 0.37<br>0.039*		
Age of HCCPs[c] (years)	**r 0.70<br><0.001***					**r 0.57<br>0.001***		**r 0.61<br><0.001***							
Women (%)														r 0.38<br>0.038*	
Female HCCPs (%)								r 0.44<br>0.013*							
Youth Index	r -0.49<br>0.005*					r -0.44<br>0.013*		r -0.37<br>0.044*					**r -0.56<br>0.001***		
Crude death rate per 100,000 inhabitants	**r 0.62<br><0.001***	r 0.37<br>0.042*				**r 0.62<br><0.001***		r 0.48<br>0.006*					r 0.47<br>0.008*		
Non-Spanish nationals (%)[b]	r -0.46<br>0.009*		**r -0.54<br>0.002***			r -0.38<br>0.037*		**r -0.56<br>0.001***							
Population aged 65 and over (%)	**r 0.66<br><0.001***					**r 0.58<br>0.001***		**r 0.51<br>0.003***		r 0.39<br>0.031*	r 0.44<br>0.013*		r 0.47<br>0.008*	r 0.36<br>0.047*	
HCCP population (%)	**r 0.72<br><0.001***	r 0.39<br>0.031*		r 0.36<br>0.048*		**r 0.71<br><0.001***	r 0.43<br>0.015*	**r 0.61<br><0.001***					r 0.38<br>0.035*	r 0.42<br>0.020*	
HCCP population <65 years (%)	**r -0.68<br><0.001***	r -0.48<br>0.006*				**r -0.50<br>0.005***		**r -0.58<br>0.001***				r -0.38<br>0.034*			
HCCP population 65-79 years (%)															
HCCP population ≥80 years (%)	**r 0.60<br><0.001***					**r 0.59<br>0.001***		**r 0.59<br><0.001***							
Level of education															
No education (%)									r 0.36<br>0.045*					r 0.38<br>0.037*	
Compulsory education (%)															
Secondary education or vocational training (%)										r -0.47<br>0.008*				r -0.43<br>0.016*	
University education (%)	r 0.42														

	0.018*														
Financial characteristics															
Mean gross annual income (euros)[b]			r 0.39<br>0.031*						r -0.36<br>0.046*	r -0.50<br>0.004*					
Mean disposable annual income (euros)[b]			r 0.37<br>0.040*						r -0.36<br>0.048*	r -0.50<br>0.005*					
Population in work in the previous four-month period (%)[b]	**r -0.52**<br>**0.003***														
Unemployed population in the previous four-month period (%)[b]															r -0.38<br>0.037*
Salaried employees (%)	r -0.48<br>0.006*											r -0.42<br>0.019*			
Self-employed (%)	r 0.48<br>0.006*											r 0.42<br>0.019*			
Agricultural sector (%)[b]										**r 0.52**<br>**0.003***					
Construction sector (%)[b]		r 0.36<br>0.047*													
Service industries (%)[b]															
Trade/Commerce (%)									r -0.41<br>0.023*						
Hospitality sector (%)[b]			r -0.49<br>0.006*			r -0.36<br>0.045*		r -0.39<br>0.031*							
Social characteristics															
No. of people assisted by Social Services [b]															
No. of families assisted by Social Services[b]							r 0.41<br>0.025*								
No. of social security benefits granted to the elderly[b]				r 0.39<br>0.032*											
No. of social security benefits granted to persons with a disability/illness[b]							r 0.37<br>0.039*								
Households with 1 person (%)					r -0.42<br>0.019*										
Households with ≥5 persons (%)			r 0.36<br>0.046*							r -0.41<br>0.024*					
Clinical characteristics (HCCP population)															
≥65 years, autonomous (%)						r -0.48<br>0.006*									
≥65 years, frail (%)						r 0.39<br>0.031*									

≥65 years, dependent (%)															
Very high complexity (Pc≥99.5) (%)															
Housebound (%)	r 0.36 0.047*														
Admitted to hospital in the previous year (%)[b]	**r -0.62 <0.001***							r -0.43 0.015*							
Seen by a specialist other than a family doctor in the previous year (%)[b]									r -0.46 0.010*					r -0.37 0.044*	
Good dietary habits (%)				r 0.39 0.028*											
Regular physical exercise (%)		r -0.37 0.048*							r -0.39 0.032*	r -0.36 0.049*	**r -0.60 0.001***				
Mean no. of visits/year to their family doctor										**r 0.57 0.001***		r 0.38 0.035*	r 0.44 0.014*		
Mean no. of visits/year to their primary care nurse									r 0.36 0.048*						
Prevalence of conditions among HCCPs (%)															
Cardiovascular conditions															
High blood pressure		**r 0.54 0.002***													
Hyperlipidaemia									r 0.40 0.026*						
Dysrhythmia[b]					r 0.43 0.017*										
Cardiac conduction disorder		r 0.38 0.035*			r 0.45 0.012*										
Ischaemic heart disease	**r -0.52 0.003***														
Heart failure															
Valvular heart disease					r 0.36 0.049*			r 0.38 0.033*							
Aortic aneurysm															
Cerebrovascular accident															r 0.47 0.008*
Endocrinal-metabolic conditions															
Diabetes mellitus															
Thyroid disorder[b]								r 0.41 0.021*						r 0.39 0.029*	
Obesity[b]	r 0.36 0.046*	r 0.43 0.015*							**r 0.52 0.003***						

Respiratory conditions														
Asthma														
Chronic obstructive pulmonary disease	r -0.38<br>0.035*					r -0.37<br>0.043*		r -0.41<br>0.022*						
Pneumonia														
Musculoskeletal conditions														
Osteoarthritis	**r 0.58**<br>**0.001***					r 0.46<br>0.009*		**r 0.60**<br>**<0.001***				r 0.40<br>0.025*		r 0.42<br>0.018*
Arthritis												r 0.44<br>0.013*		
Osteoporosis		**r 0.51**<br>**0.004***						**r 0.59**<br>**0.001***						
Nervous system/neurodegenerative conditions														
Dementia[b]		**r 0.57**<br>**0.001***				r 0.47<br>0.008*		**r 0.53**<br>**0.002***			r 0.49<br>0.005*			r 0.41<br>0.021*
Parkinson's[b]														
Epilepsy					**r 0.55**<br>**0.001***		r 0.38<br>0.035*							
Paralysis					r 0.47<br>0.008*		r 0.38<br>0.034*			r -0.49<br>0.005*				
Mental health conditions														
Depression			r 0.48<br>0.006*											r 0.39<br>0.031*
Anxiety				r 0.36<br>0.045*										r 0.46<br>0.009*
Schizophrenia	r 0.43<br>0.016*					r 0.39<br>0.030*				r 0.46<br>0.010*		r 0.39<br>0.029*	r 0.43<br>0.016*	
Alcohol use disorder														
Substance abuse[b]														
Suicidal behaviour or suicide attempt			**r 0.55**<br>**0.001***		r 0.37<br>0.043*									
Liver/kidney conditions														
Chronic renal failure														
Liver disease[b]					r -0.37<br>0.040*									
Oncological conditions														
Prior neoplasm														
Active neoplasm[b]			r -0.46<br>0.010*											

Metastasis											r -0.38<br>0.037*	r -0.44<br>0.014*			
Non-Hodgkin lymphoma		r 0.36<br>0.044*													
Other															
Urinary tract infection	**r 0.55**<br>**0.002***	r 0.40<br>0.024*				**r 0.58**<br>**0.001***		r 0.49<br>0.005*							
Septicaemia[b]	r 0.43<br>0.016*														
Lupus			r 0.37<br>0.042*												
Glaucoma															
Anaemia	**r 0.58**<br>**0.001***					r 0.41<br>0.021*									
Pharmacological treatments among HCCPs (%)															
Analgesics										**r 0.65**<br>**<0.001***	r 0.36<br>0.049*				
Antidepressants	r 0.37<br>0.041*		r 0.40<br>0.025*			r 0.46<br>0.009*		**r 0.51**<br>**0.003***			r 0.43<br>0.016*			r 0.46<br>0.010*	r 0.37<br>0.043*
Anxiolytics										r 0.38<br>0.034*					
Opioids															
Hypnotics/sedatives															
Antipsychotics	r 0.47<br>0.007*					**r 0.57**<br>**0.001***	**r 0.55**<br>**0.001***	r 0.44<br>0.013*						r 0.36<br>0.044*	
Anti-dementia drugs						**r 0.62**<br>**<0.001***		**r 0.65**<br>**<0.001***			r 0.40<br>0.025*				r 0.42<br>0.020*

[a] Nursing diagnoses by NANDA-I code: Toileting self-care deficit [00110]; Chronic low self-esteem [00119]; Situational low self-esteem [00120]; Hopelessness [00124]; Powerlessness [00125]; Chronic confusion [00129]; Disturbed thought processes [00130]; Impaired memory [00131]; Complicated grieving [00135]; Grieving [00136]; Chronic sorrow [00137]; Fear [00148]; Risk for Situational low self-esteem [00153]; Sedentary lifestyle [00168]; Risk for acute confusion [00173]. [b] Non-normally distributed variable. [c] HCCPS: highly complex chronic patients. [d] r, Pearson's or Spearman's correlation coefficient, as appropriate. * Statistically significant p-value. In bold, correlation coefficient values between 0.51-0.75 (moderate/strong association). In bold and italics, correlation coefficient values between 0.76-1.00 (strong/perfect association).

Table S1. Correlations between the prevalence of NANDA-I nursing diagnoses and socio-demographic, financial, and clinical characteristics (V).

Characteristics	Nursing diagnoses by NANDA-I code [a]									
	**00188**	**00198**	**00204[b]**	**00206[b]**	**00231[b]**	**00232[b]**	**00233[b]**	**00239[b]**	**00249**	**00257[b]**
Basic population characteristics										
Population (no. of inhabitants)[b]		r -0.39<br>0.030*	r 0.40<br>0.026*							
Age (years)	**r[d] 0.64<br><0.001***	**r 0.59<br>0.001***			**r 0.60<br><0.001***		r 0.38<br>0.035*		**r 0.64<br><0.001***	
Age of HCCPs[c] (years)	r 0.45<br>0.011*	**r 0.64<br><0.001***			**r 0.61<br><0.001***				**r 0.66<br><0.001***	
Women (%)		r 0.36<br>0.045*								
Female HCCPs (%)										
Youth Index	**r -0.55<br>0.001***	r -0.37<br>0.040*			**r -0.61<br><0.001***		r -0.43<br>0.016*		r -0.42<br>0.019*	r -0.39<br>0.029*
Crude death rate per 100,000 inhabitants	**r 0.57<br>0.001***	r 0.47<br>0.007*			**r 0.66<br><0.001***			r 0.43<br>0.016*	**r 0.56<br>0.001***	r 0.44<br>0.013*
Non-Spanish nationals (%)[b]									r -0.47<br>0.007*	
Population aged 65 and over (%)	**r 0.67<br>< 0.001***	**r 0.61<br><0.001***		r -0.37<br>0.043*	**r 0.59<br><0.001***		r 0.38<br>0.038*	r 0.36<br>0.050*	**r 0.62<br><0.001***	
HCCP population (%)	r 0.49<br>0.005*	r 0.47<br>0.007*			**r 0.56<br>0.001***				**r 0.60<br><0.001***	
HCCP population <65 years (%)	r -0.38<br>0.036*	**r -0.55<br>0.001***			**r -0.51<br>0.003***				**r -0.55<br>0.001***	
HCCP population 65-79 years (%)					r -0.42<br>0.018*					**r -0.65<br><0.001***
HCCP population ≥80 years (%)	r 0.38<br>0.036*	**r 0.57<br>0.001***			**r 0.59<br>0.001***				**r 0.58<br>0.001***	
Level of education										
No education (%)					r -0.36<br>0.050*			r 0.43<br>0.017*		r -0.43<br>0.015*
Compulsory education (%)										

Secondary education or vocational training (%)	r -0.49 0.005*									
University education (%)										
Financial characteristics										
Mean gross annual income (euros)[b]	r -0.44 0.012*			r 0.42 0.018*				r -0.48 0.007*		
Mean disposable annual income (euros)[b]	r -0.44 0.012*			r 0.45 0.012*				r -0.46 0.010*		
Population in work in the previous four-month period (%)[b]										
Unemployed population in the previous four-month period (%)[b]							r -0.37 0.042*			
Salaried employees (%)										
Self-employed (%)										
Agricultural sector (%)[b]	**r 0.57 0.001***	r 0.39 0.031*		r -0.36 0.046*				r 0.36 0.047*	r 0.38 0.034*	
Construction sector (%)[b]										
Service industries (%)[b]										
Trade/Commerce (%)										
Hospitality sector (%)[b]										
Social characteristics										
No. of people assisted by Social Services [b]			**r 0.51 0.003***							
No. of families assisted by Social Services[b]			r 0.39 0.034*							
No. of social security benefits granted to the elderly[b]			r 0.44 0.014*							
No. of social security benefits granted to persons with a disability/illness[b]			r 0.38 0.036*							
Households with 1 person (%)										
Households with ≥5 persons (%)			r 0.37 0.039*			r -0.47 0.007*				
Clinical characteristics (HCCP population)										
≥65 years, autonomous (%)										
≥65 years, frail (%)										
≥65 years, dependent (%)										r 0.46 0.010*
Very high complexity (Pc≥99.5) (%)										
Housebound (%)		r 0.40 0.028*								

Admitted to hospital in the previous year (%)[b]				r 0.42<br>0.017*			r 0.40<br>0.027*			
Seen by a specialist other than a family doctor in the previous year (%)[b]	r -0.41<br>0.021*			r 0.48<br>0.006*						**r 0.58**<br>**0.001***
Good dietary habits (%)									r 0.44<br>0.013*	
Regular physical exercise (%)							r -0.45<br>0.012*	r -0.41<br>0.027*		
Mean no. of visits/year to their family doctor						r 0.37<br>0.040*				
Mean no. of visits/year to their primary care nurse										
Prevalence of conditions among HCCPs (%)										
Cardiovascular conditions										
High blood pressure										
Hyperlipidaemia										
Dysrhythmia[b]									r 0.38<br>0.033*	
Cardiac conduction disorder										
Ischaemic heart disease				r 0.36<br>0.044*					r -0.50<br>0.004*	
Heart failure										
Valvular heart disease				r -0.42<br>0.019*						
Aortic aneurysm										
Cerebrovascular accident										
Endocrinal-metabolic conditions										
Diabetes mellitus										
Thyroid disorder[b]										
Obesity[b]	r 0.37<br>0.041*									
Respiratory conditions										
Asthma	**r 0.52**<br>**0.003***				r 0.39<br>0.032*				**r 0.52**<br>**0.003***	
Chronic obstructive pulmonary disease								r 0.37<br>0.039*		
Pneumonia										
Musculoskeletal conditions										
Osteoarthritis									r 0.46<br>0.010*	
Arthritis										

Osteoporosis									
Nervous system/neurodegenerative conditions									
Dementia[b]									
Parkinson's[b]									
Epilepsy									
Paralysis								r -0.47 0.008*	
Mental health conditions									
Depression									
Anxiety									
Schizophrenia					r 0.40 0.028*			r 0.38 0.037*	
Alcohol use disorder				r 0.47 0.008*					
Substance abuse[b]									
Suicidal behaviour or suicide attempt									
Liver/kidney conditions									
Chronic renal failure			r -0.40 0.028*						
Liver disease[b]		r -0.38 0.035*		**r 0.53 0.002***					
Oncological conditions									
Prior neoplasm									
Active neoplasm[b]						r 0.49 0.006*			
Metastasis									
Non-Hodgkin lymphoma		r 0.42 0.018*						r -0.38 0.035*	
Other									
Urinary tract infection	**r 0.62 <0.001***	**r 0.52 0.002***		r -0.49 0.006*					**r 0.55 0.001***
Septicaemia[b]						r -0.43 0.016*			
Lupus									
Glaucoma				**r 0.54 0.002***				r -0.39 0.031*	
Anaemia									**r 0.51 0.003***
Pharmacological treatments among HCCPs (%)									

Analgesics				r 0.38<br>0.036*	
Antidepressants					r 0.38<br>0.035*
Anxiolytics	r 0.36<br>0.044*				
Opioids					
Hypnotics/sedatives					
Antipsychotics					r 0.38<br>0.035*
Anti-dementia drugs					

[a] Nursing diagnoses by NANDA-I code: Risk-prone health behavior [00188]; Disturbed sleep pattern [00198]; Ineffective peripheral tissue perfusion [00204]; Risk for bleeding [00206]; Risk for frail elderly syndrome [00231]; Obesity [00232]; Overweight [00233]; Risk for impaired cardiovascular function [00239]; Risk for pressure ulcer [00249]; Frail elderly syndrome [00257]. [b] Non-normally distributed variable. [c] HCCPS: highly complex chronic patients. [d] r: Pearson's or Spearman's correlation coefficient, as appropriate. * Statistically significant p-value. In bold, correlation coefficient values between 0.51-0.75 (moderate/strong association). In bold and italics, correlation coefficient values between 0.76-1.00 (strong/perfect association).

Figure S1. Municipalities in the healthcare area under study distributed in metropolitan, northern, and southern areas.

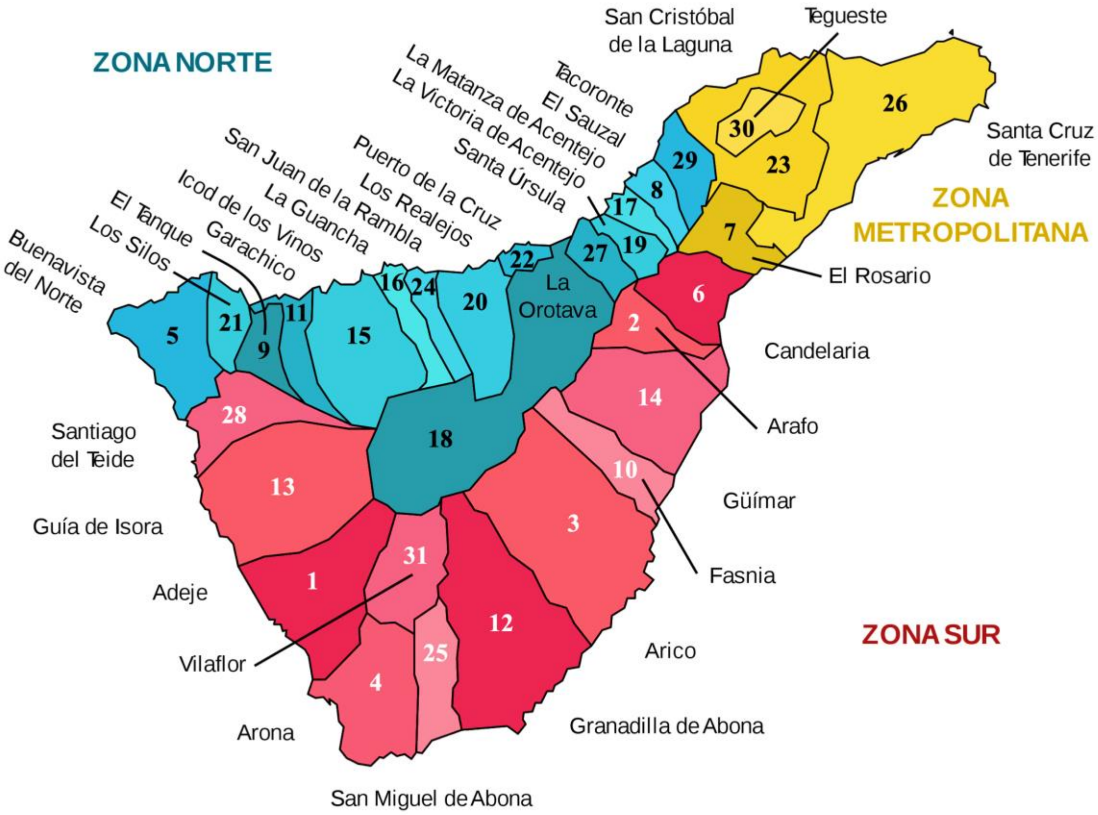

Figure S2. Prevalence (%) of population dysfunctionality by municipality in the healthcare area under study. Health pattern 1 - Health perception/Health management.

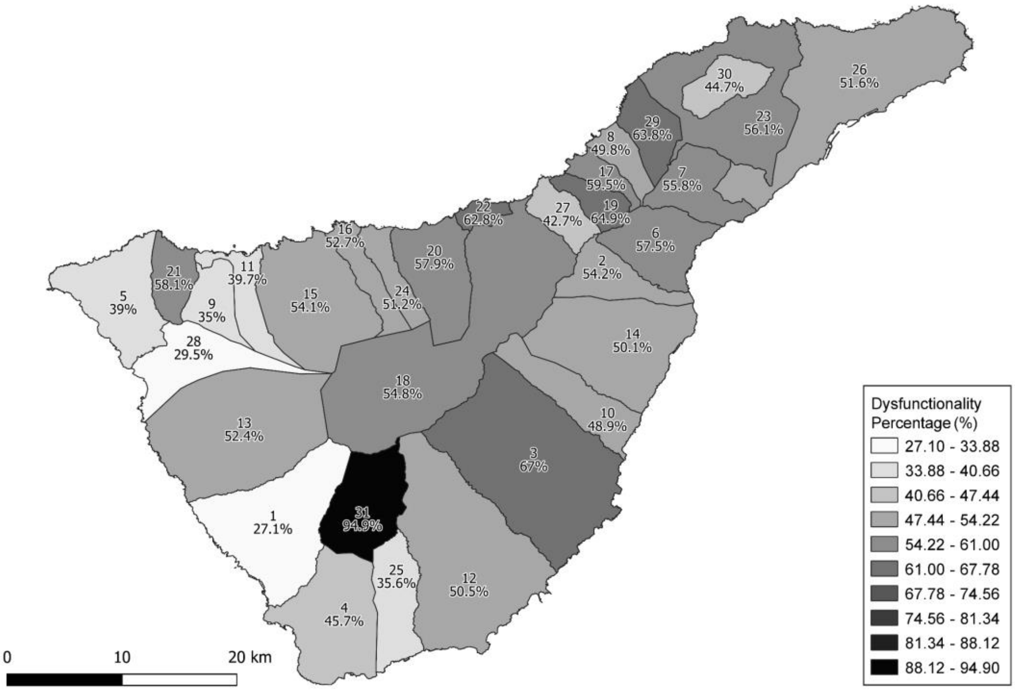

Figure S3. Prevalence (%) of population dysfunctionality by municipality in the healthcare area under study. Health pattern 2 - Nutrition/Metabolism.

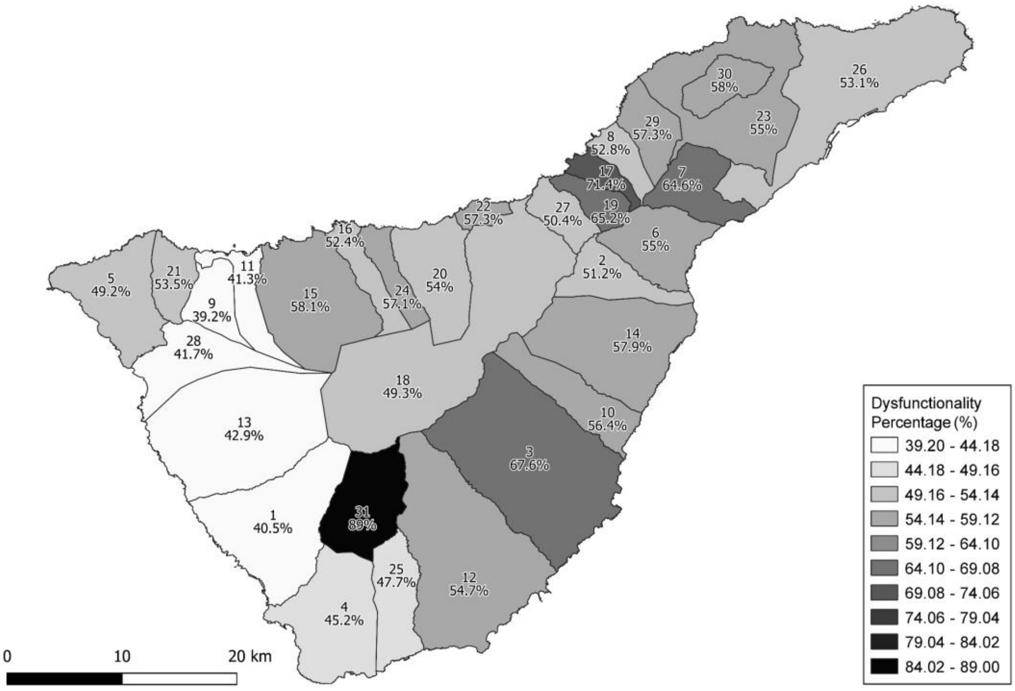

Figure S4. Prevalence (%) of population dysfunctionality by municipality in the healthcare area under study. Health pattern 3 - Elimination.

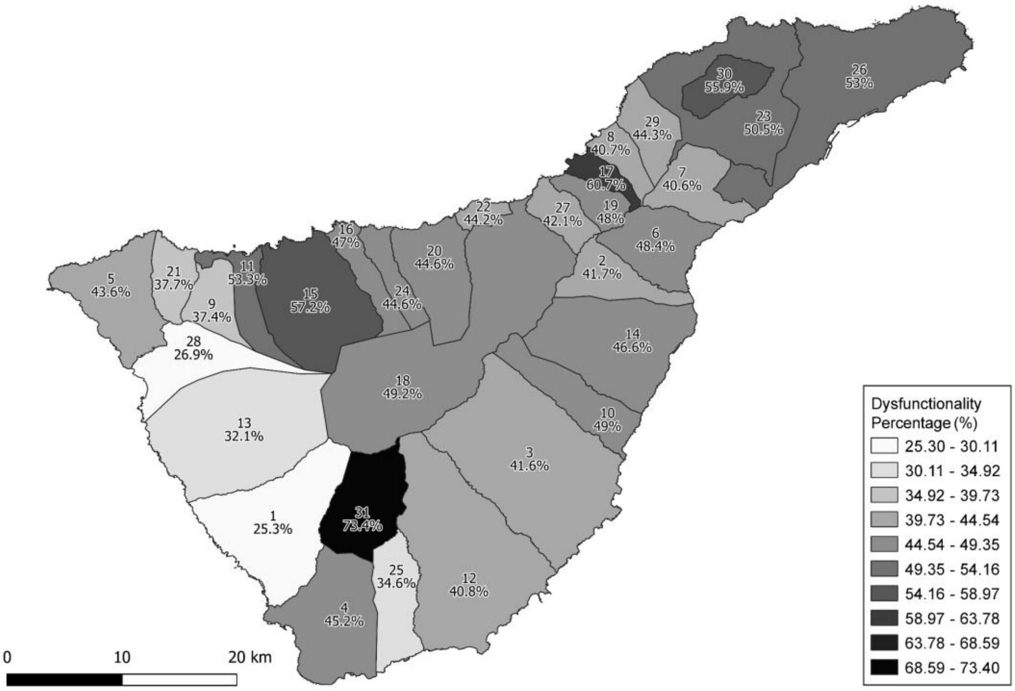

Figure S5. Prevalence (%) of population dysfunctionality by municipality in the healthcare area under study. Health pattern 4 - Physical activity/Exercise.

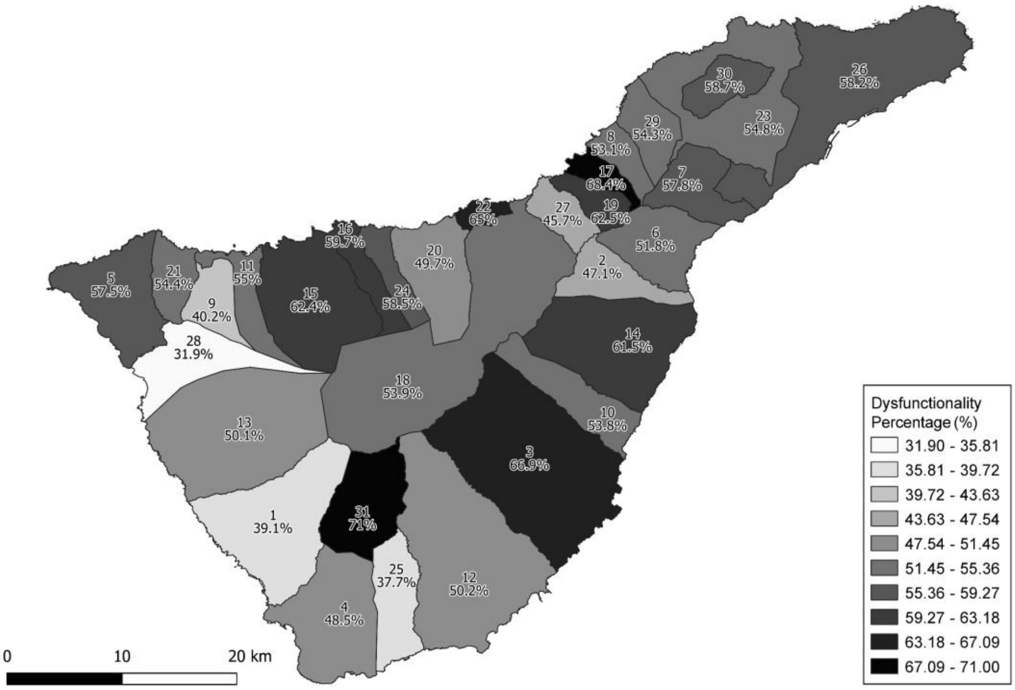

Figure S6. Prevalence (%) of population dysfunctionality by municipality in the healthcare area under study. Health pattern 5 - Sleep/Rest.

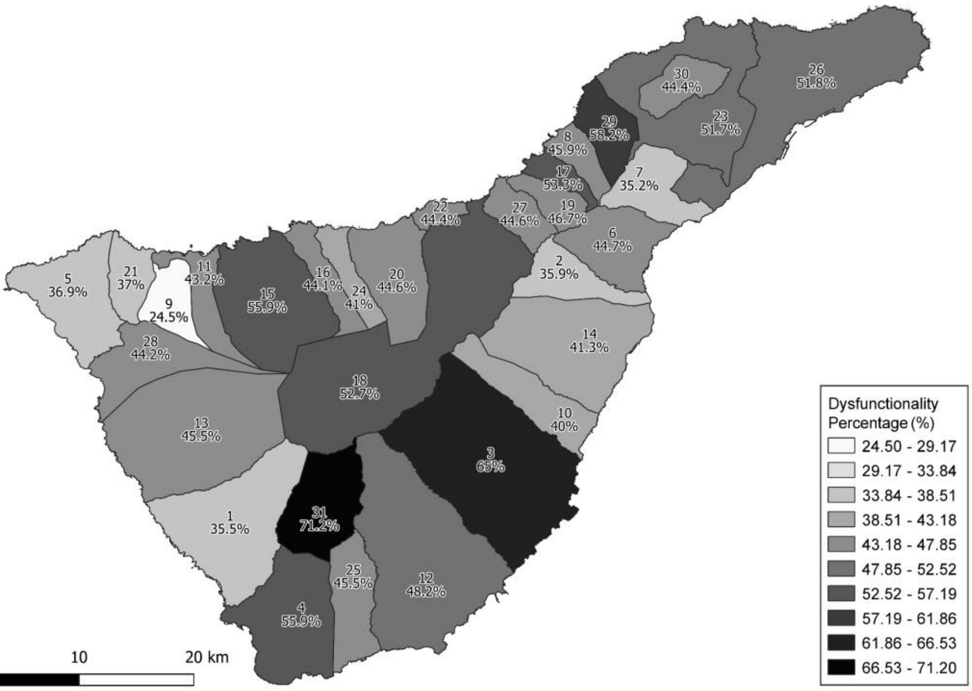

Figure S7. Prevalence (%) of population dysfunctionality by municipality in the healthcare area under study. Health pattern 6 - Cognition/Perception.

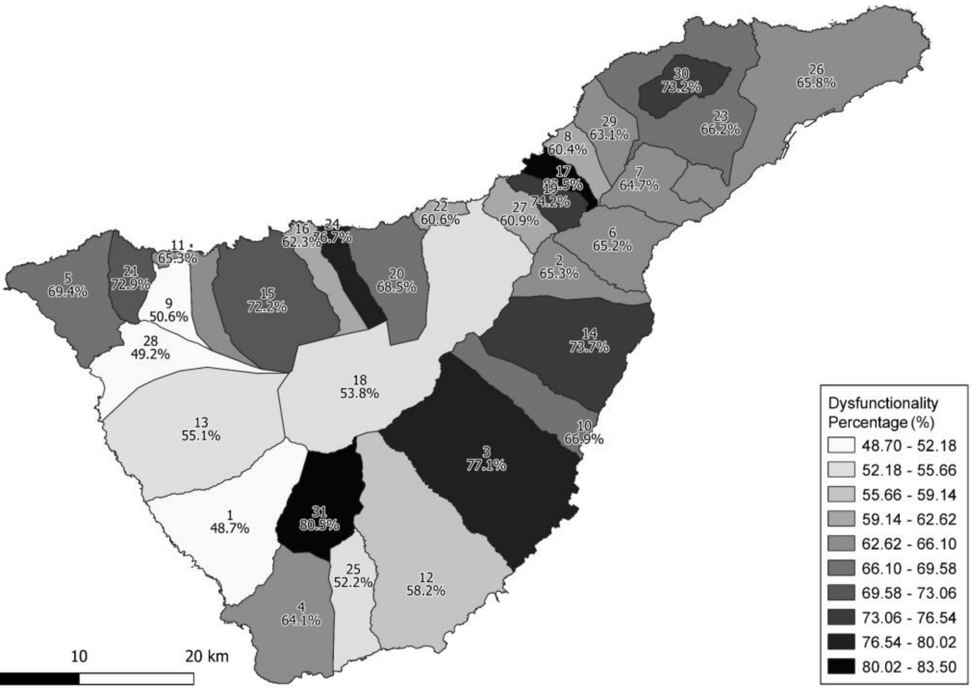

Figure S8. Prevalence (%) of population dysfunctionality by municipality in the healthcare area under study. Health pattern 7 - Self-perception/Self-concept.

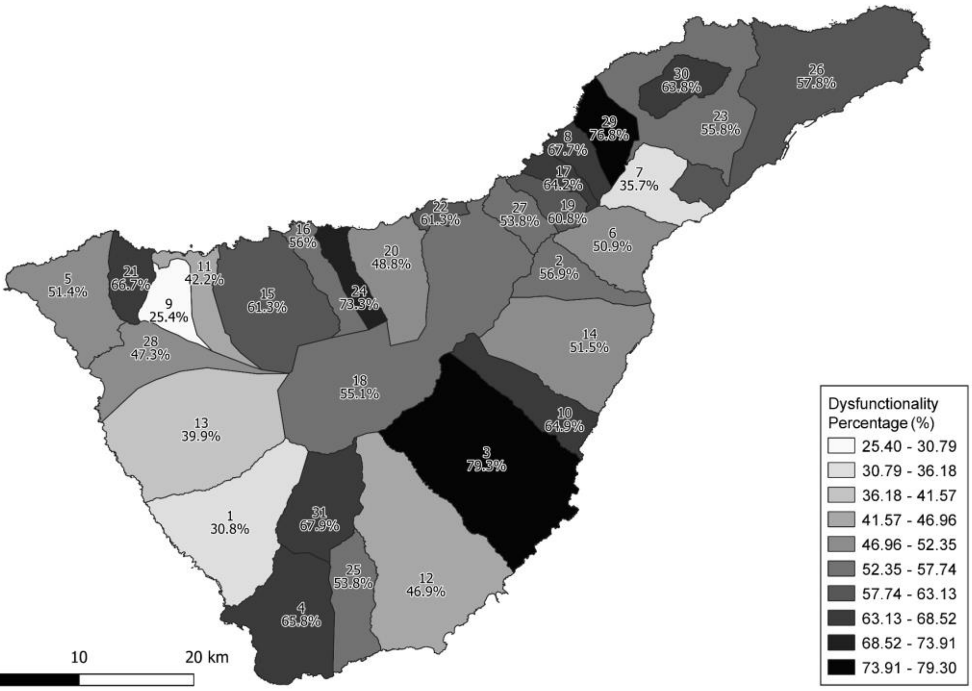

Figure S9. Prevalence (%) of population dysfunctionality by municipality in the healthcare area under study. Health pattern 8 - Role/Relationships.

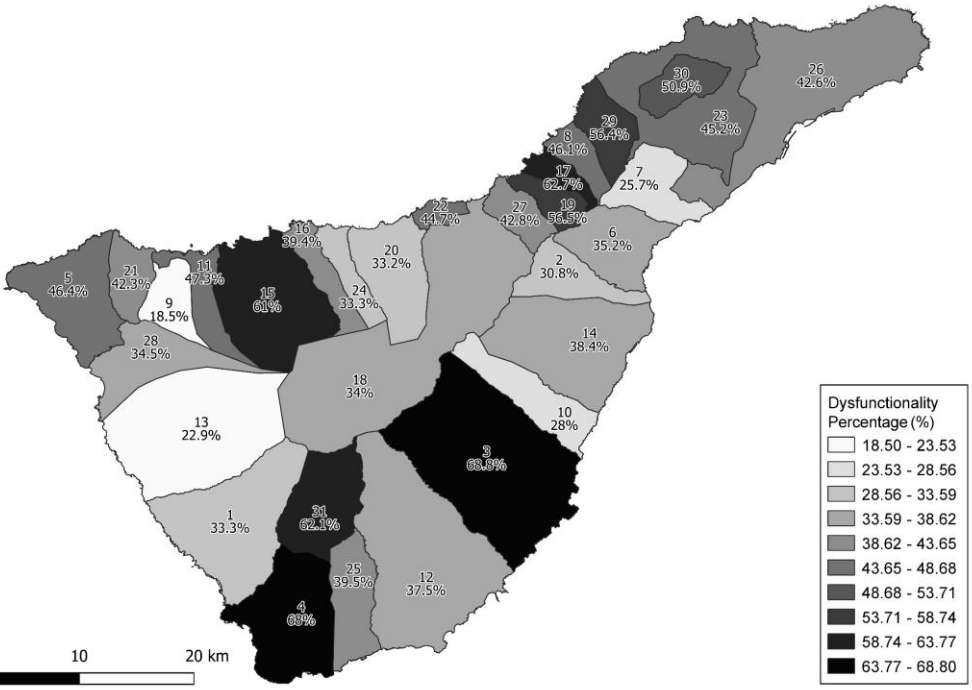

Figure S10. Prevalence (%) of population dysfunctionality by municipality in the healthcare area under study. Health pattern 9 - Sexuality/Reproduction.

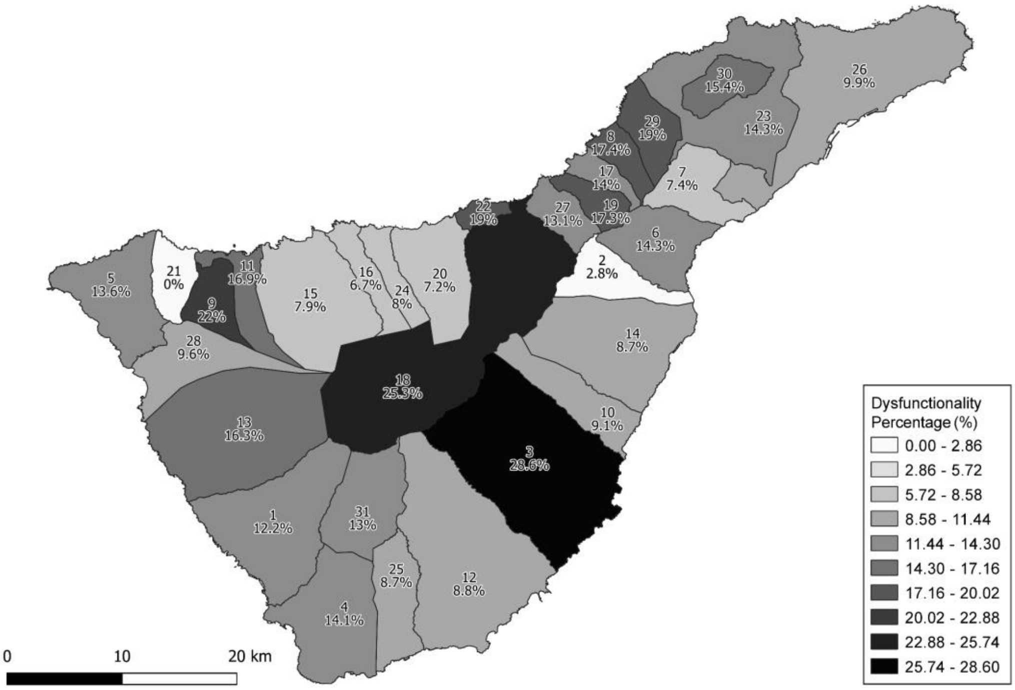

Figure S11. Prevalence (%) of population dysfunctionality by municipality in the healthcare area under study. Health pattern 10 - Coping/Stress tolerance.

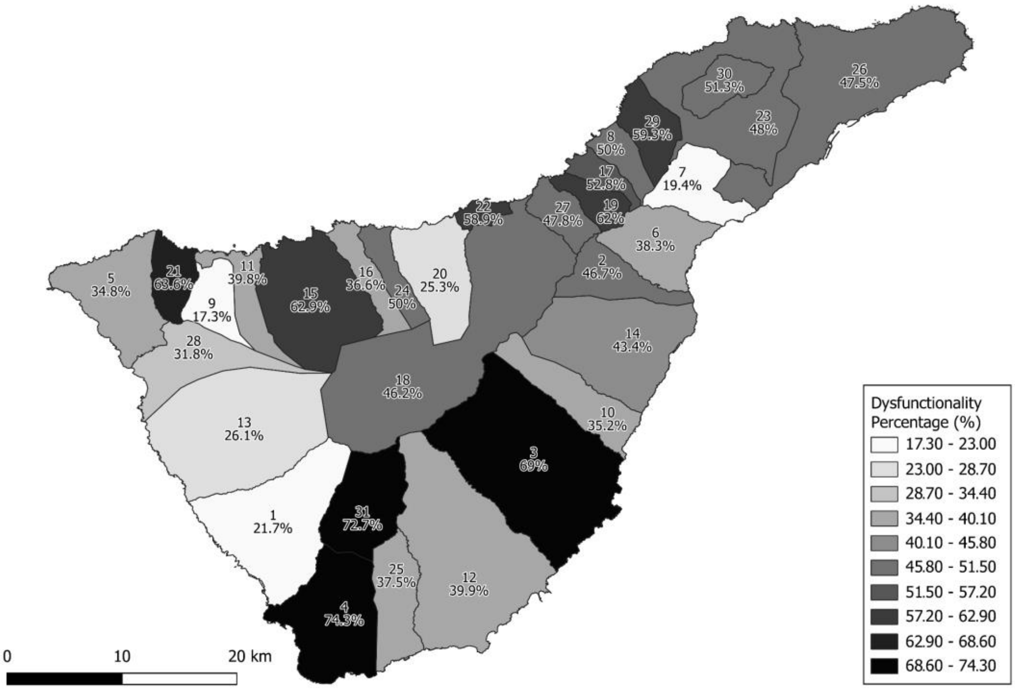

Figure S12. Prevalence (%) of population dysfunctionality by municipality in the healthcare area under study. Health pattern 11 - Values/beliefs.

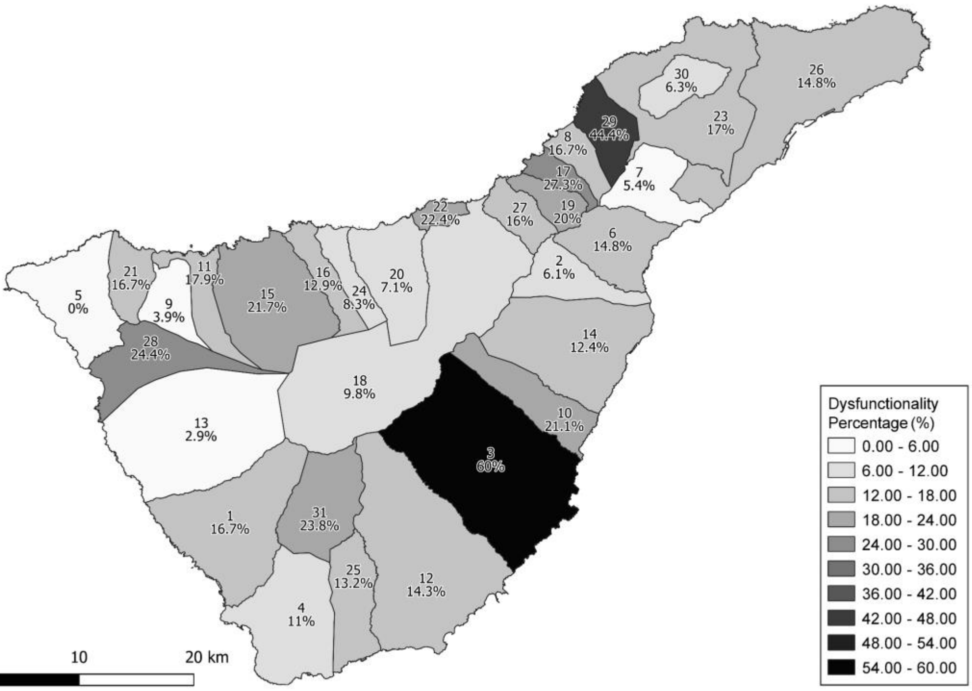

Figure S13. Population prevalence (%) of the nursing diagnosis Readiness for enhanced health management [NANDA-I 00162] by municipalities in the healthcare area under study.

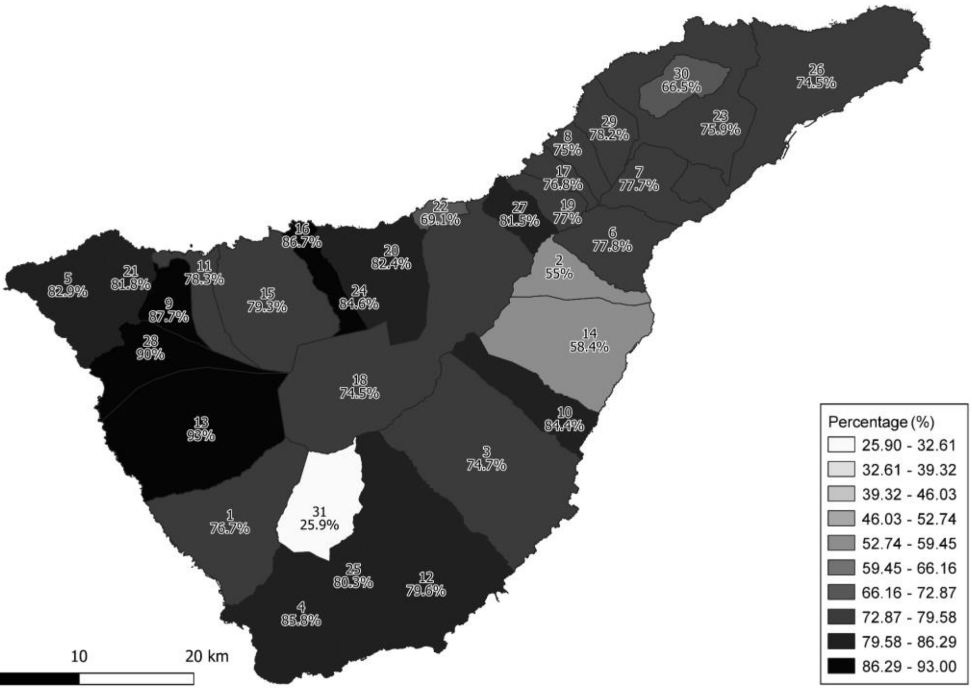

Figure S14. Population prevalence (%) of the nursing diagnosis Willingness to improve immunization status [NANDA-I 00186] by municipalities in the healthcare area under study.

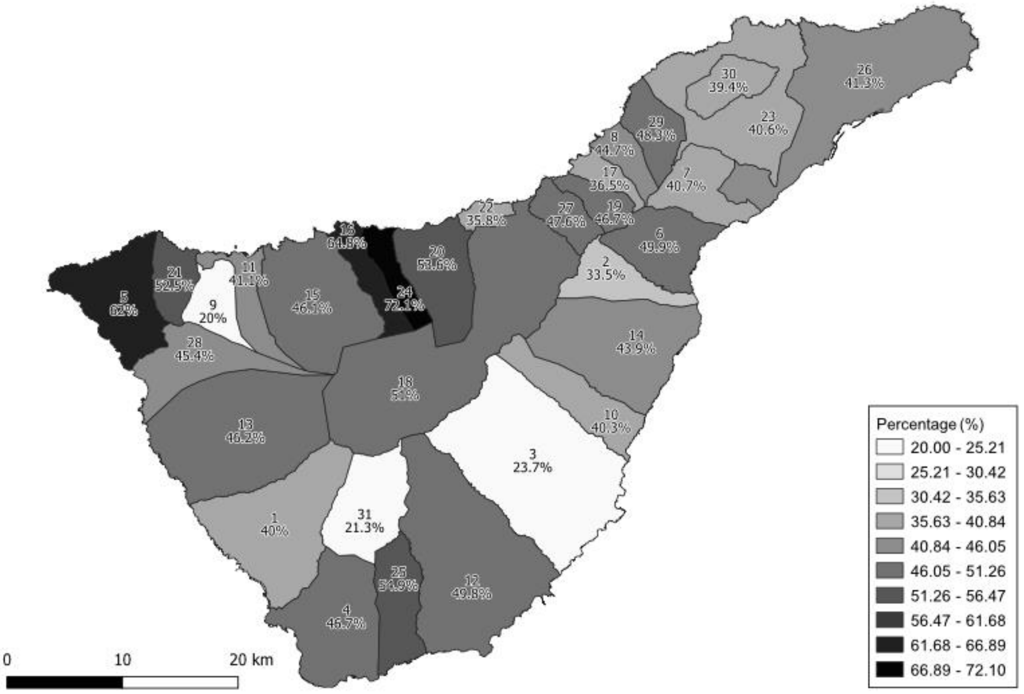

Figure S15. Population prevalence (%) of the nursing diagnosis Impaired skin integrity [NANDA-I 00046] by municipalities in the healthcare area under study.

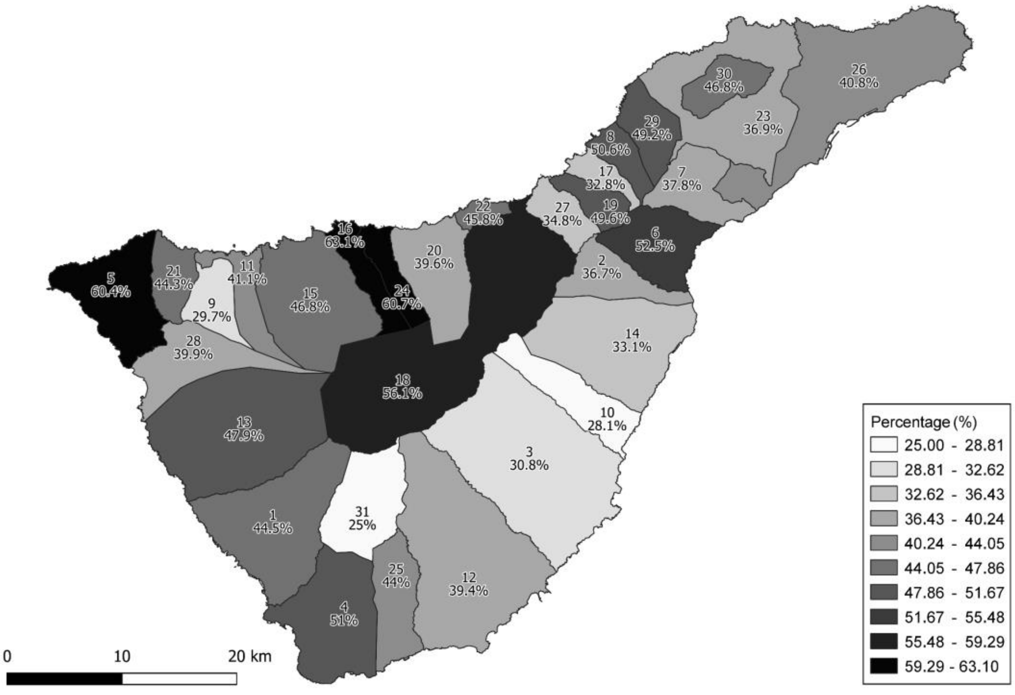

Figure S16. Population prevalence (%) of the nursing diagnosis Acute pain [NANDA-I 00132] by municipalities in the healthcare area under study.

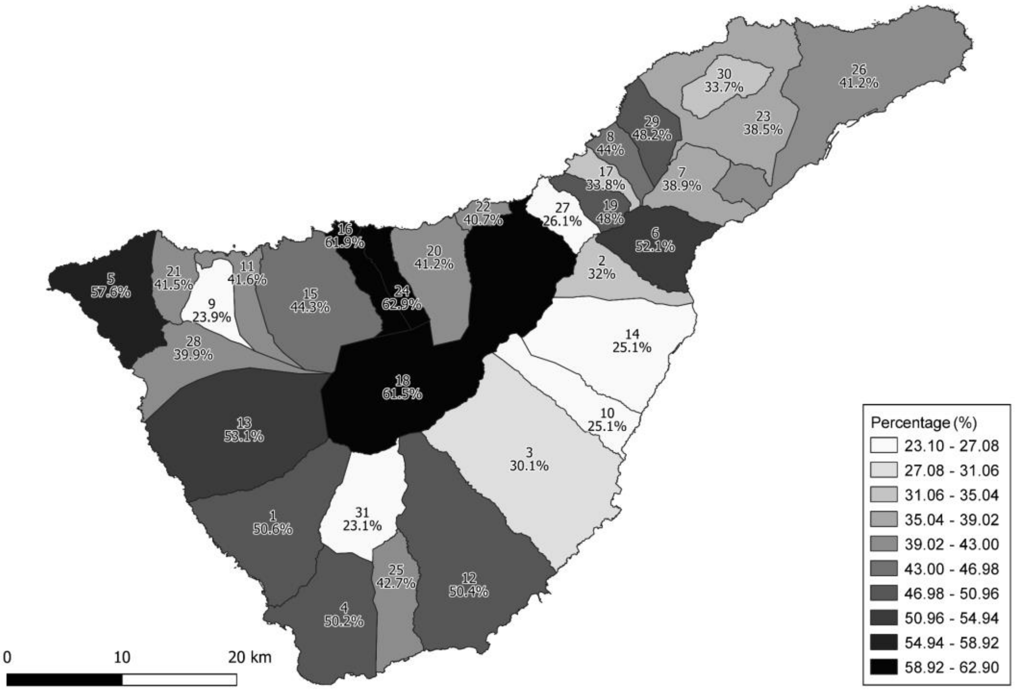

Figure S17. Population prevalence (%) of the nursing diagnosis Impaired comfort [NANDA-I 00214] by municipalities in the healthcare area under study.

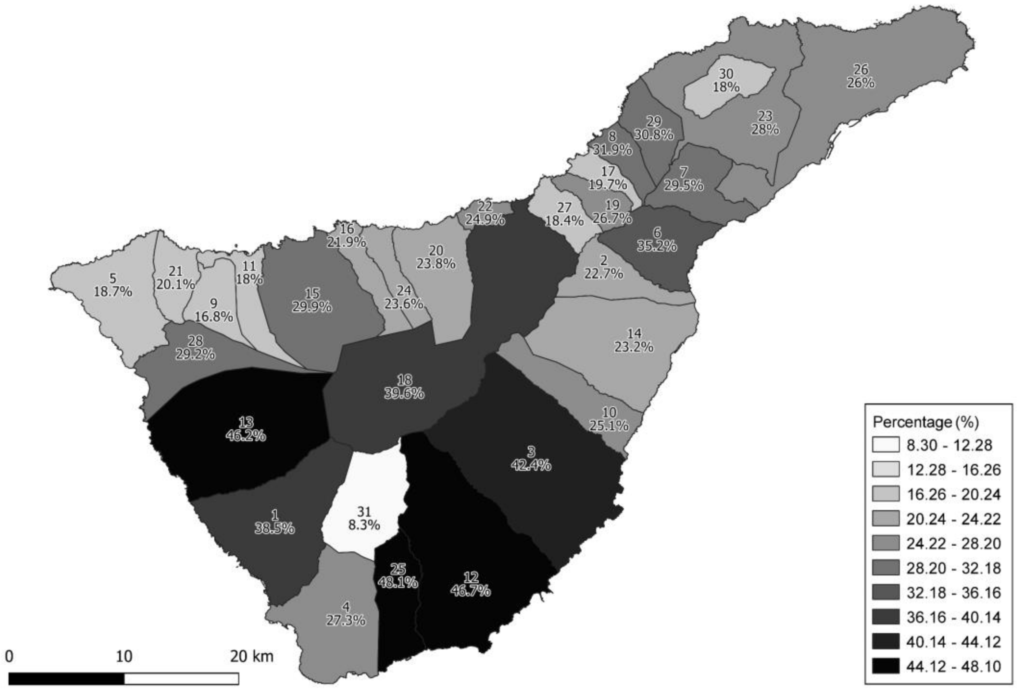

Figure S18. Population prevalence (%) of the nursing diagnosis Ineffective protection [NANDA-I 00043] by municipalities in the healthcare area under study.

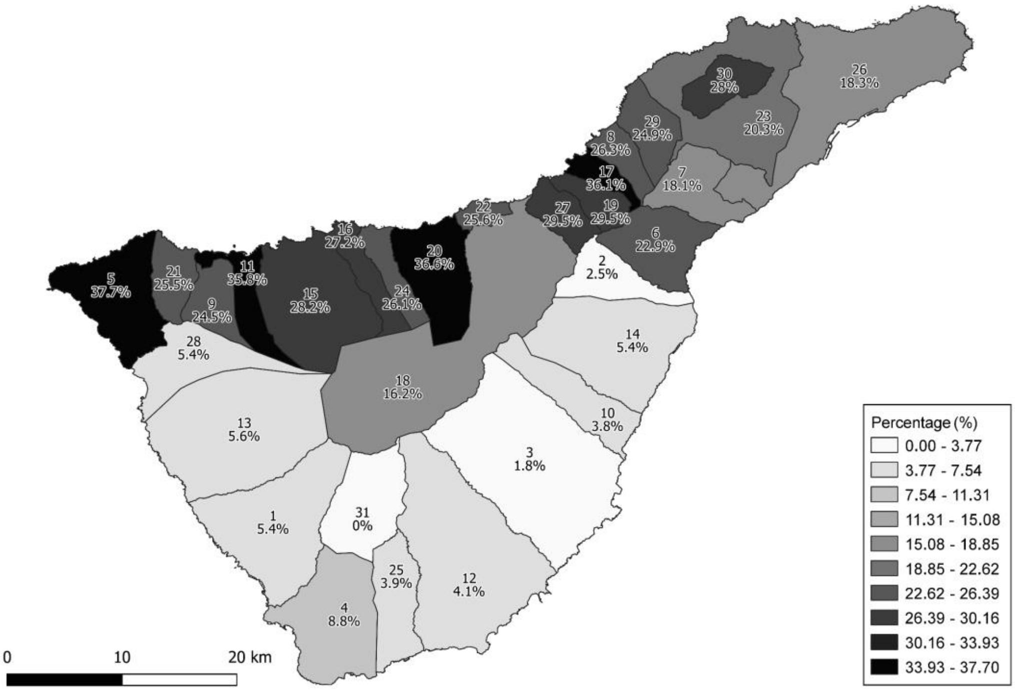

Figure S19. Population prevalence (%) of the nursing diagnosis Ineffective breathing pattern [NANDA-I 00032] by municipalities in the healthcare area under study.

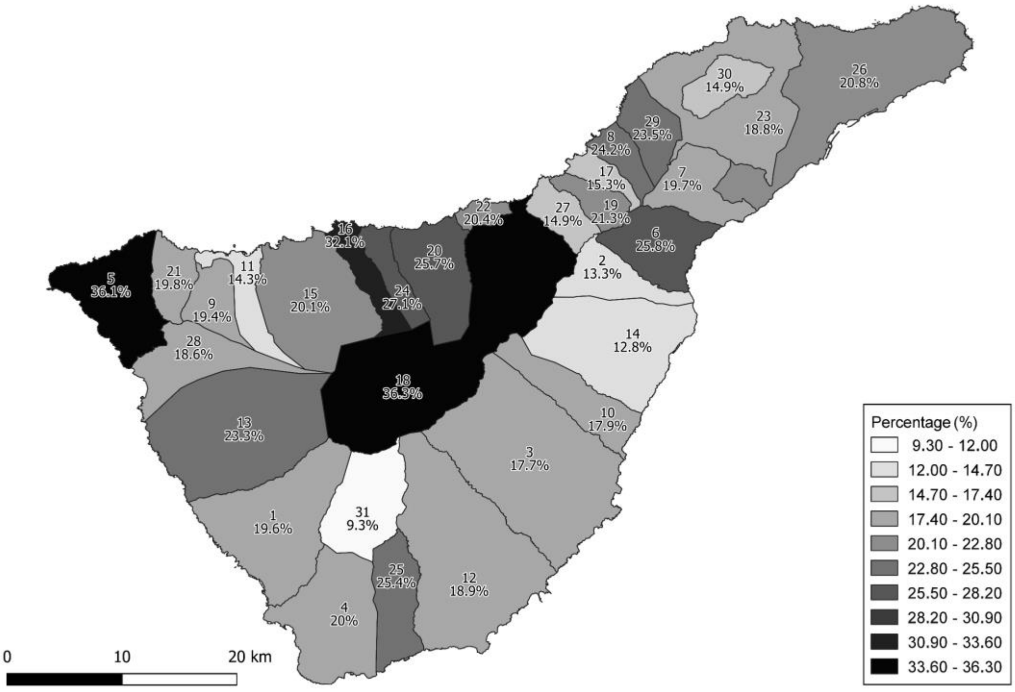

Figure S20. Population prevalence (%) of the nursing diagnosis Risk for falls [NANDA-I 00155] by municipalities in the healthcare area under study.

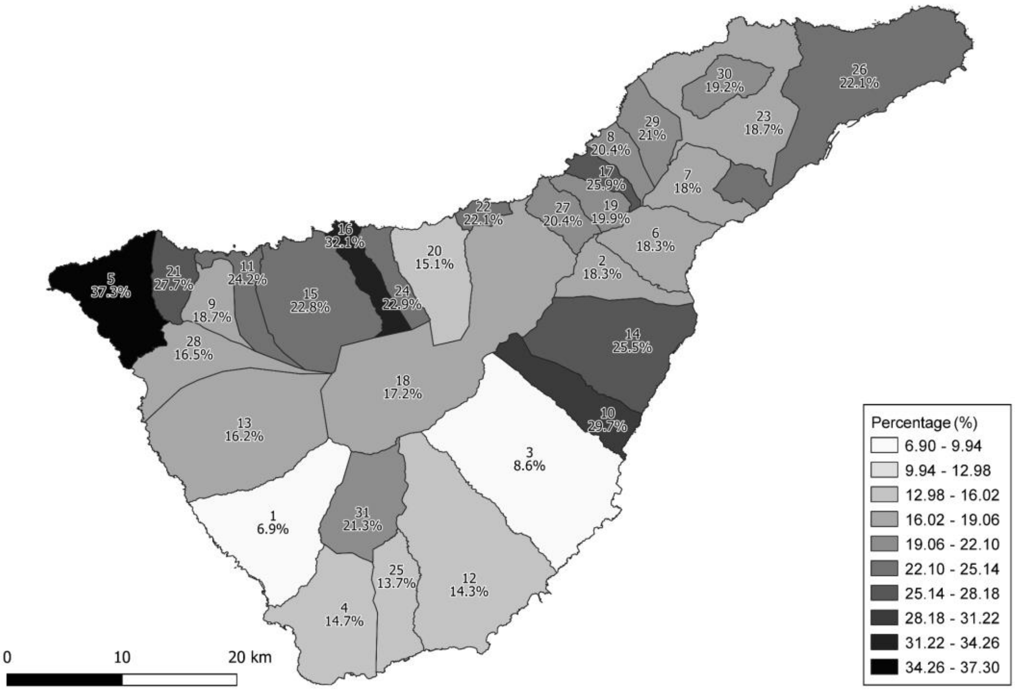

Figure S21. Population prevalence (%) of the nursing diagnosis Impaired urinary elimination [NANDA-I 00016] by municipalities in the healthcare area under study.

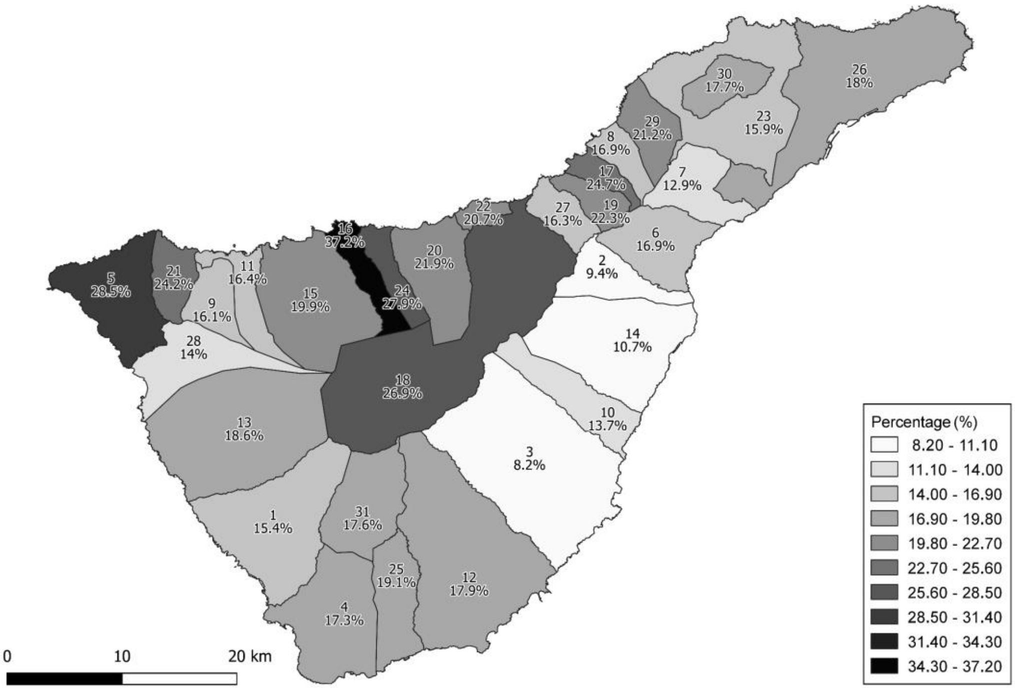

Figure S22. Population prevalence (%) of the nursing diagnosis Risk for unstable blood glucose level [NANDA-I 00179] by municipalities in the healthcare area under study.

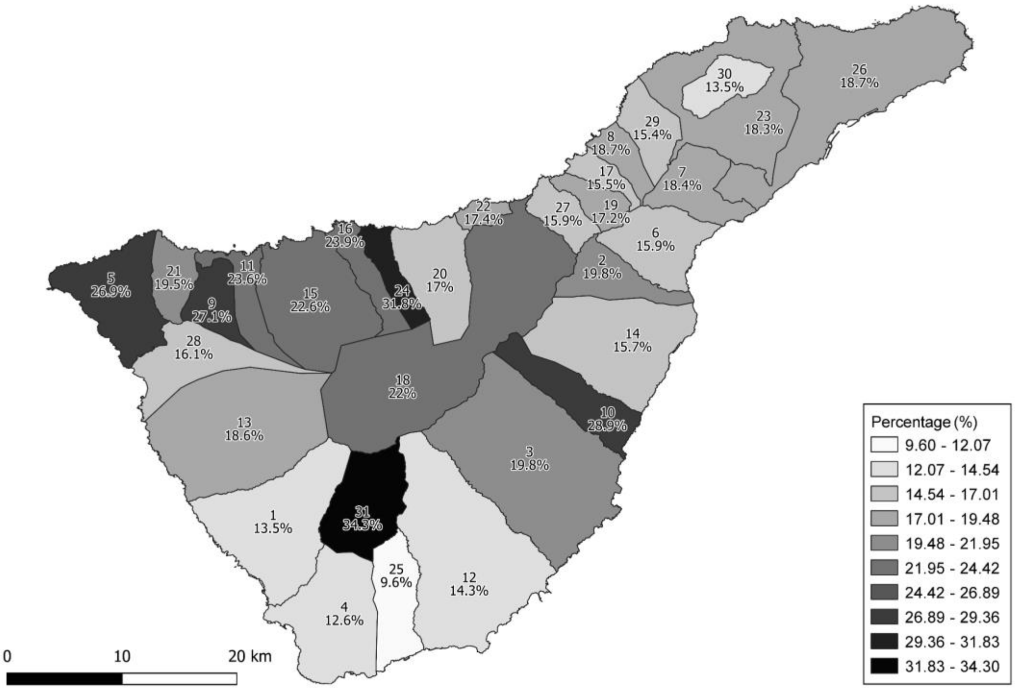

Figure S23. Population prevalence (%) of the nursing diagnosis Nutritional imbalance: excess [NANDA-I 00001] by municipalities in the healthcare area under study.

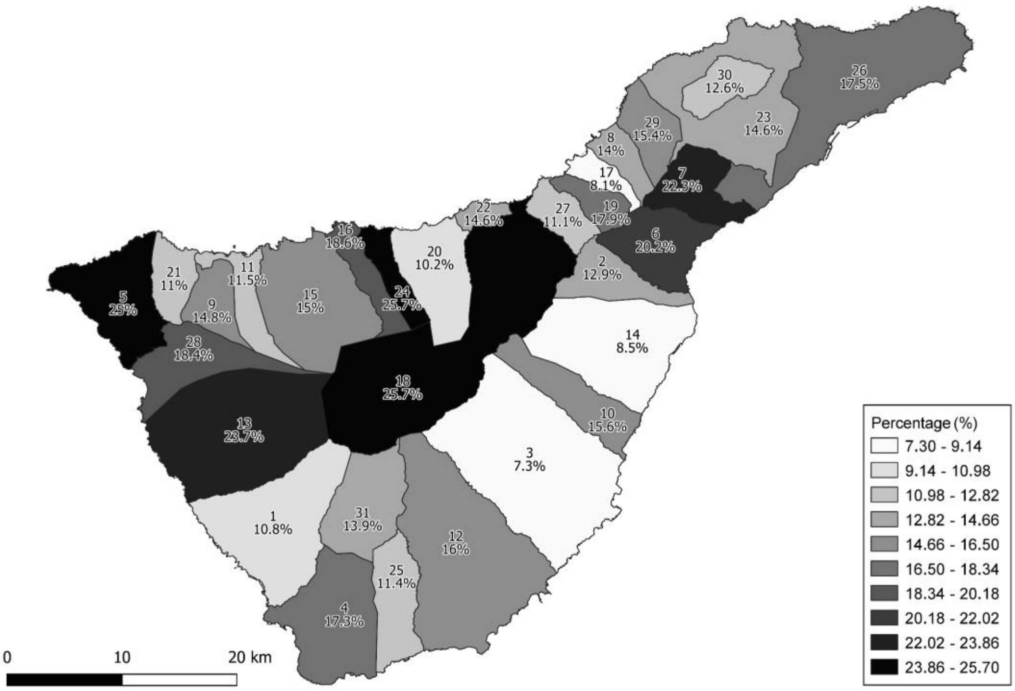

Figure S24. Population prevalence (%) of the nursing diagnosis Chronic pain [NANDA-I 00133] by municipalities in the healthcare area under study.

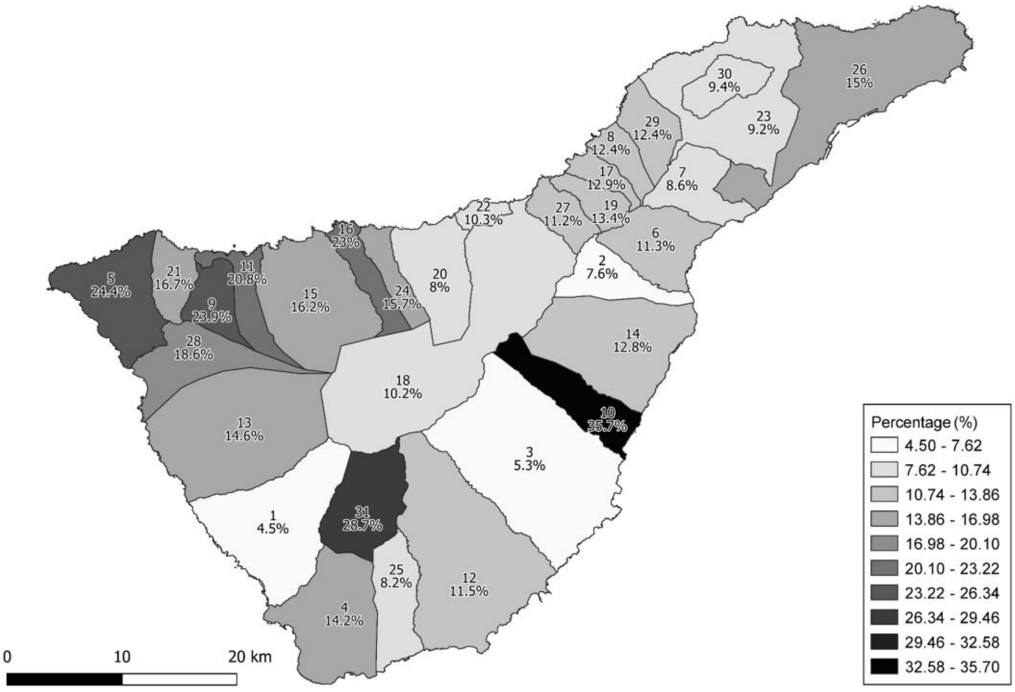

Figure S25. Population prevalence (%) of the nursing diagnosis Ineffective health management [NANDA-I 00078] by municipalities in the healthcare area under study.

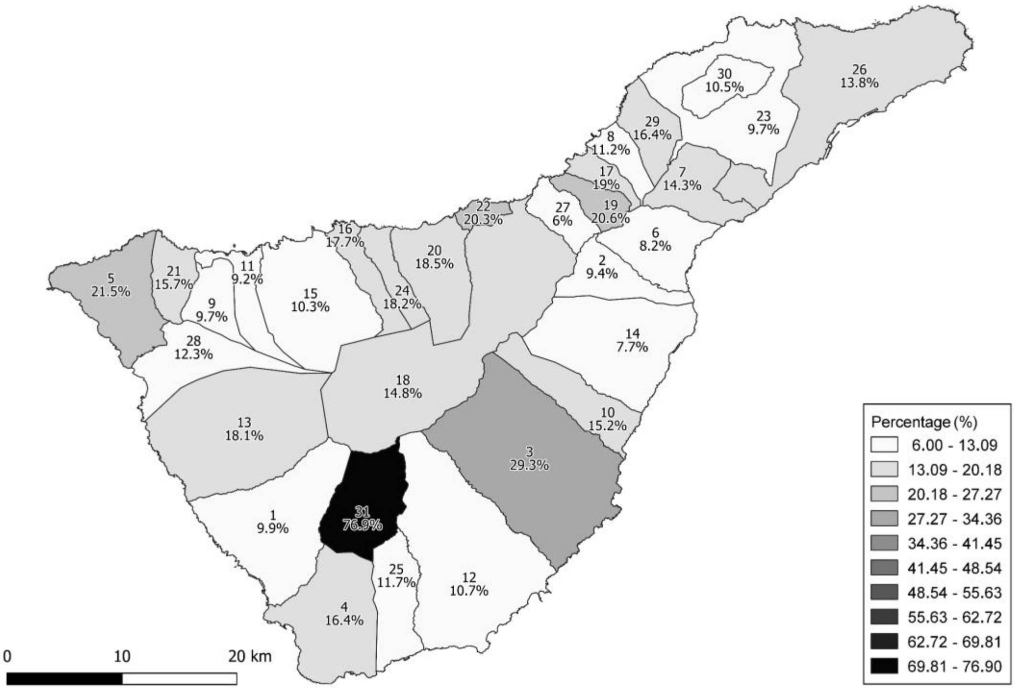

Figure S26. Population prevalence (%) of the nursing diagnosis Risk for infection [NANDA-I 00004] by municipalities in the healthcare area under study.

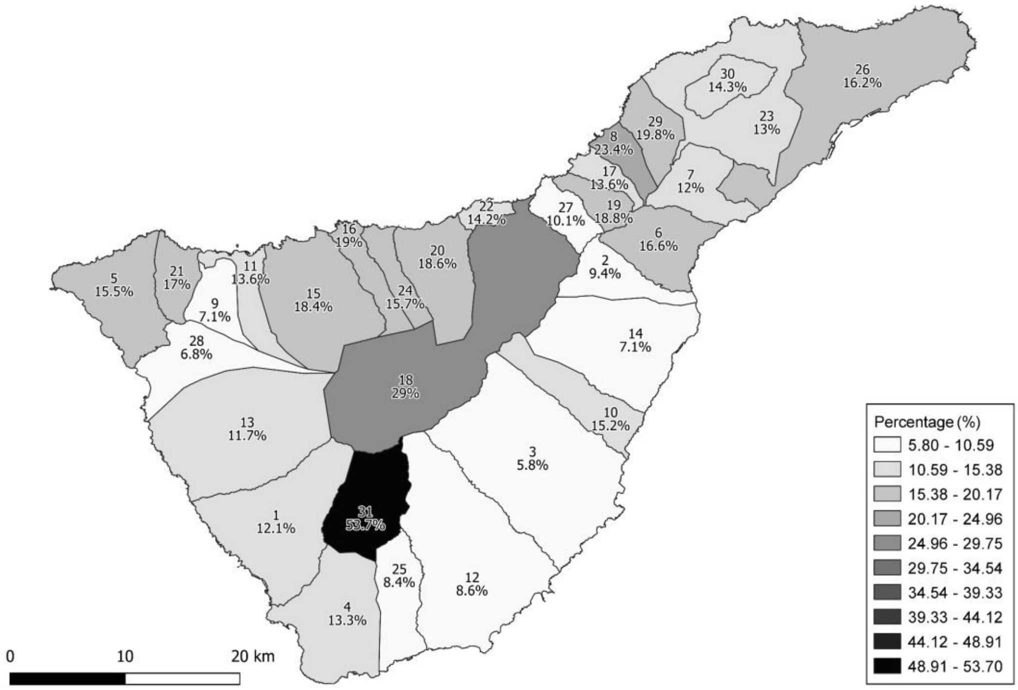

Figure S27. Population prevalence (%) of the nursing diagnosis Risk for injury [NANDA-I 00035] by municipalities in the healthcare area under study.

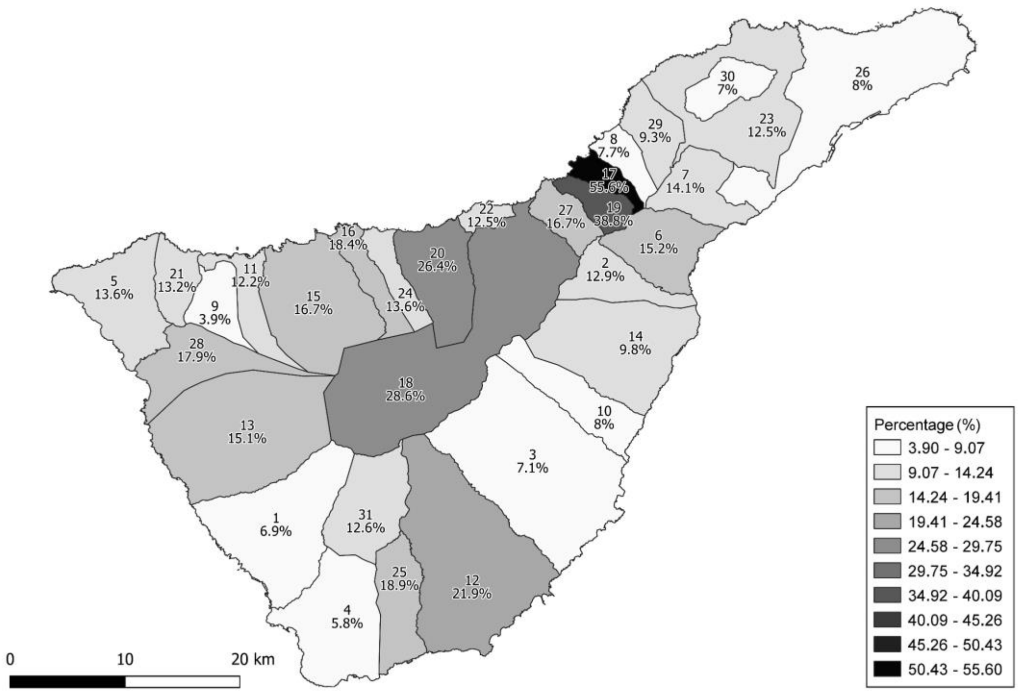

Figure S28. Population prevalence (%) of the nursing diagnosis Anxiety [NANDA-I 00146] by municipalities in the healthcare area under study.

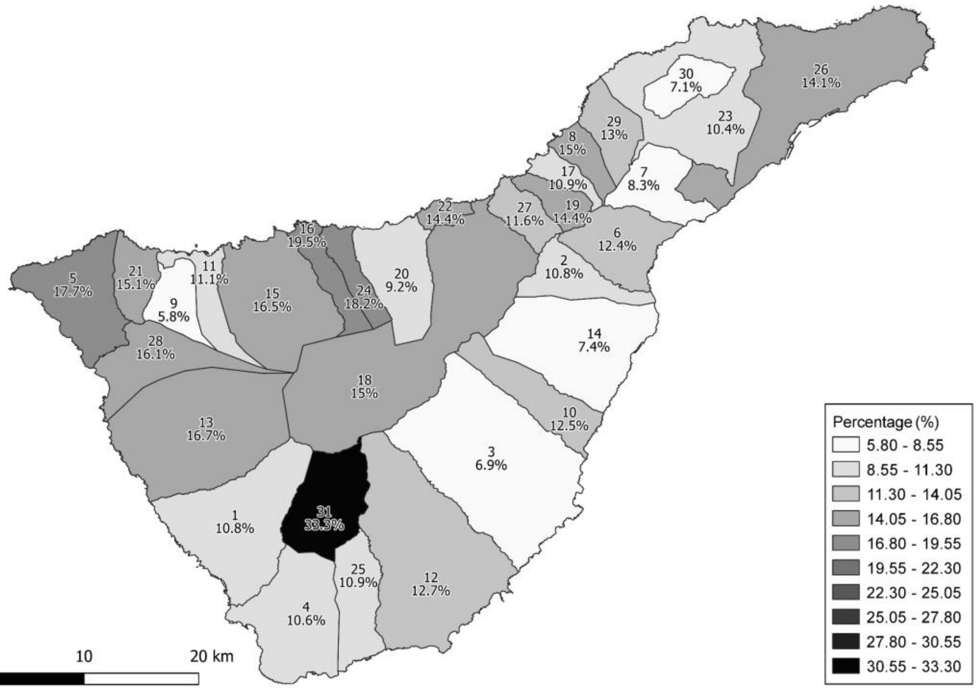

Figure S29. Population prevalence (%) of the nursing diagnosis Health-generating behaviors [NANDA-I 00084] by municipalities in the healthcare area under study.

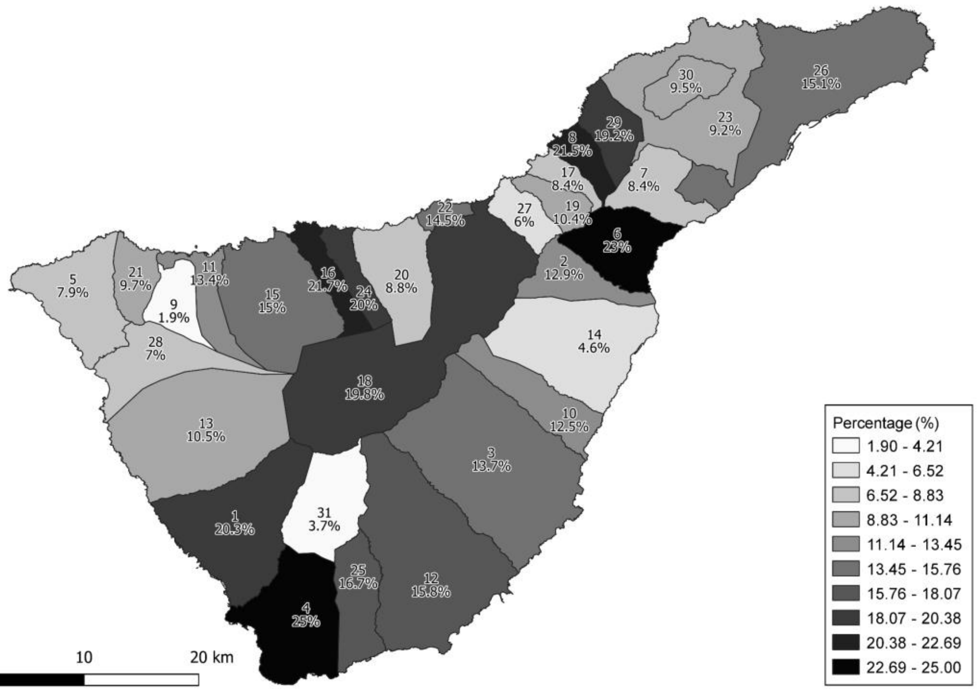

Figure S30. Population prevalence (%) of the nursing diagnosis Noncompliance [NANDA-I 00079] by municipalities in the healthcare area under study.

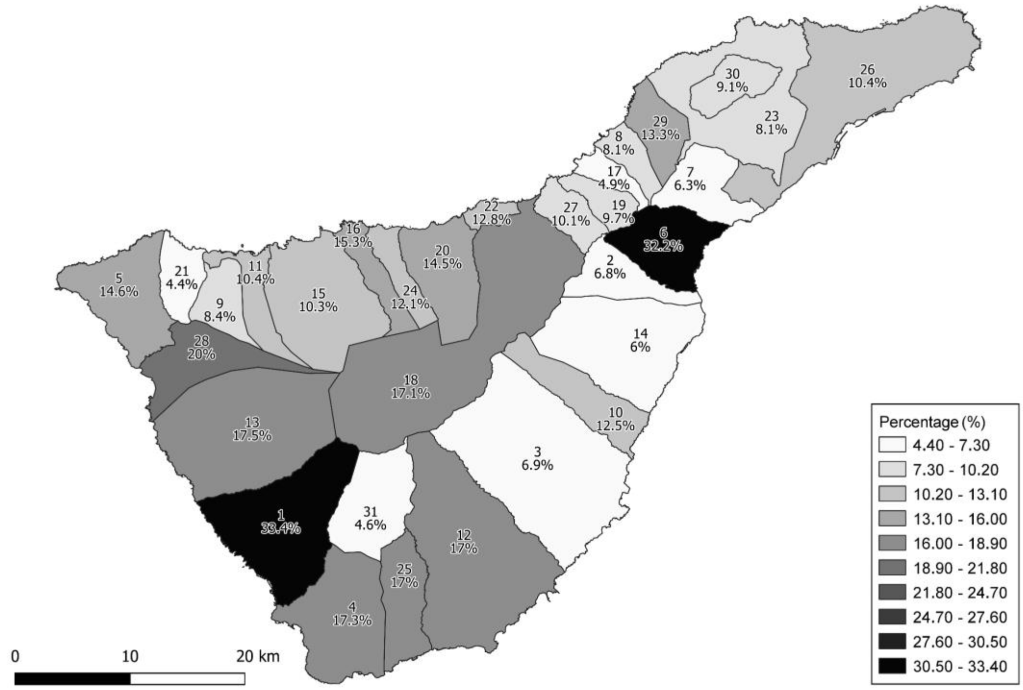

Figure S31. Population prevalence (%) of the nursing diagnosis Bathing self-care deficit [NANDA-I 00108] by municipalities in the healthcare area under study.

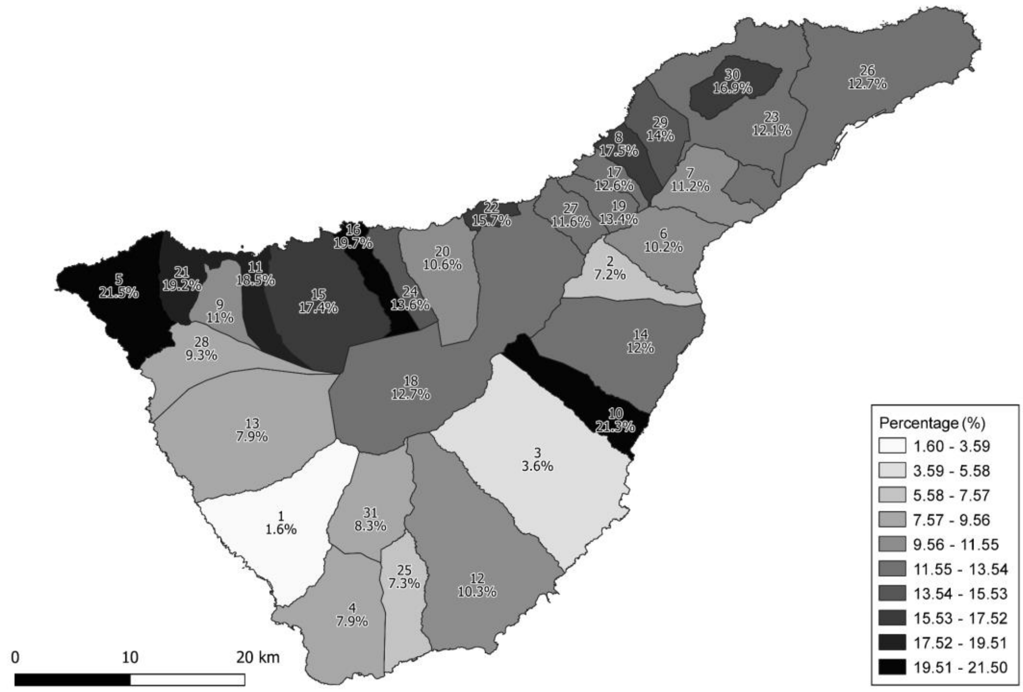

Figure S32. Population prevalence (%) of the nursing diagnosis Sensory perception disturbance: visual, auditory, kinaesthetic, tactile [NANDA-I 00122] by municipalities in the healthcare area under study.

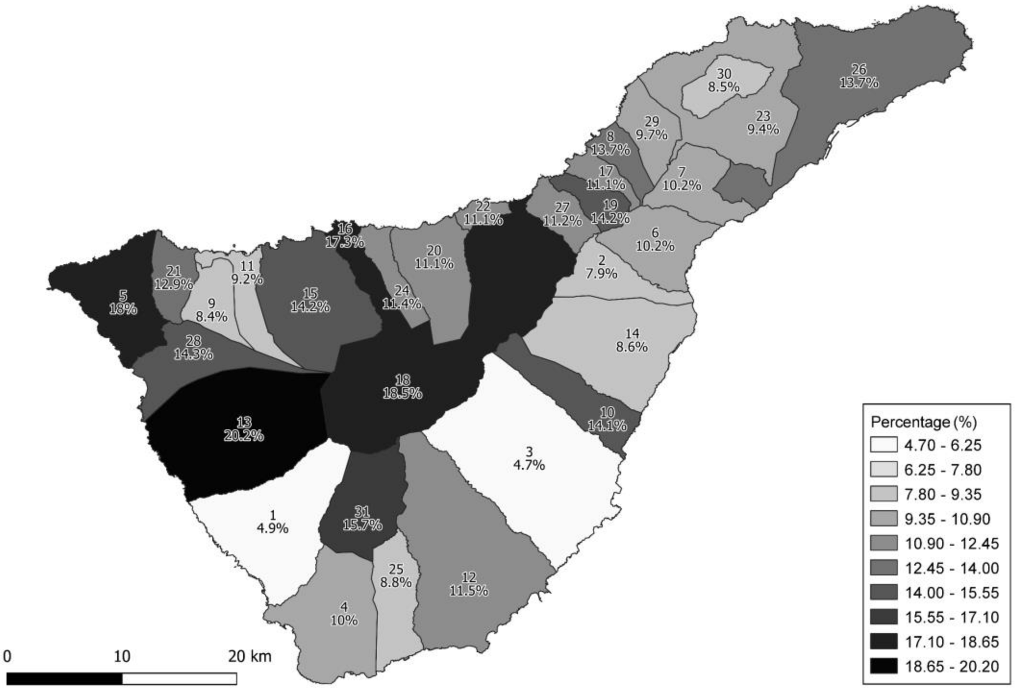

Figure S33. Population prevalence (%) of the nursing diagnosis Impaired walking [NANDA-I 00088] by municipalities in the healthcare area under study.

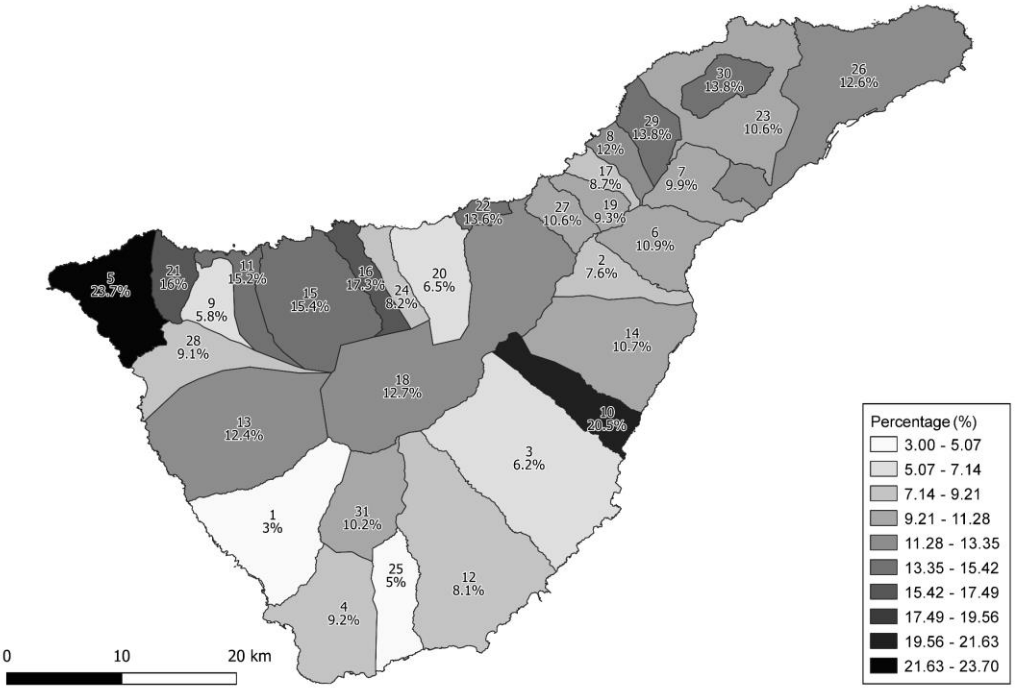

Figure S34. Population prevalence (%) of the nursing diagnosis Impaired physical mobility [NANDA-I 00085] by municipalities in the healthcare area under study.

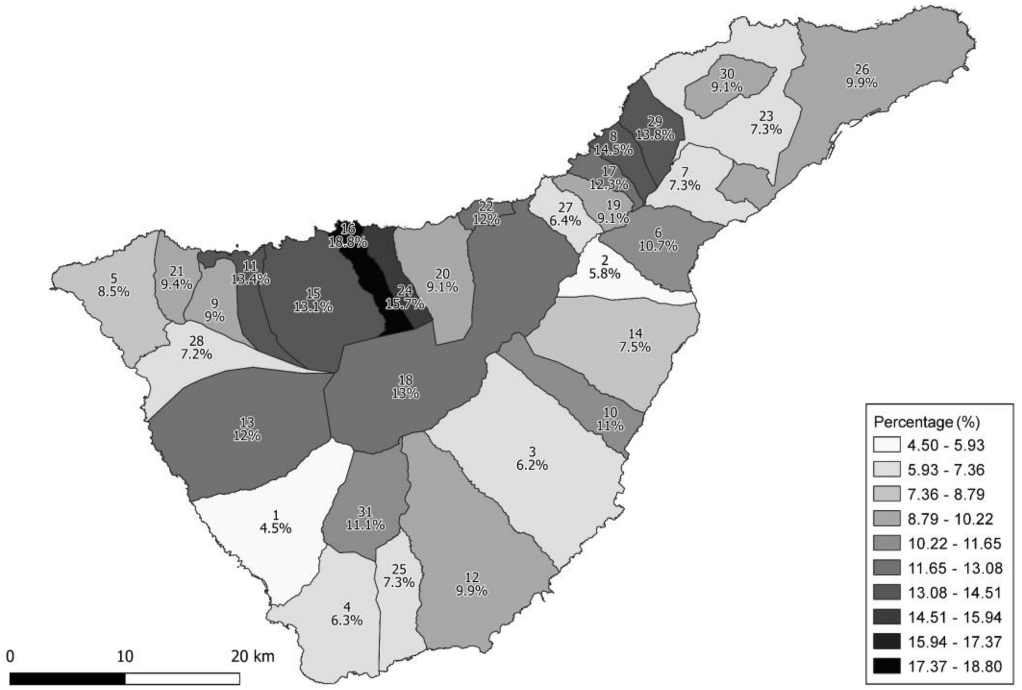

Figure S35. Population prevalence (%) of the nursing diagnosis Dressing self-care deficit [NANDA-I 00109] by municipalities in the healthcare area under study.

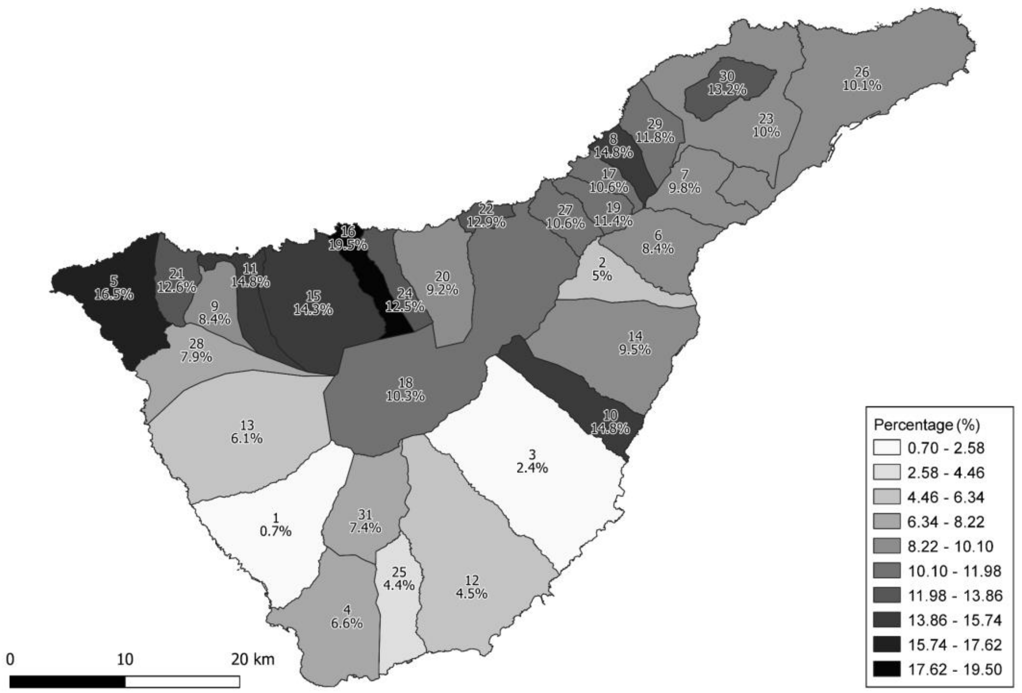

Figure S36. Population prevalence (%) of the nursing diagnosis Ineffective health maintenance [NANDA-I 00099] by municipalities in the healthcare area under study.

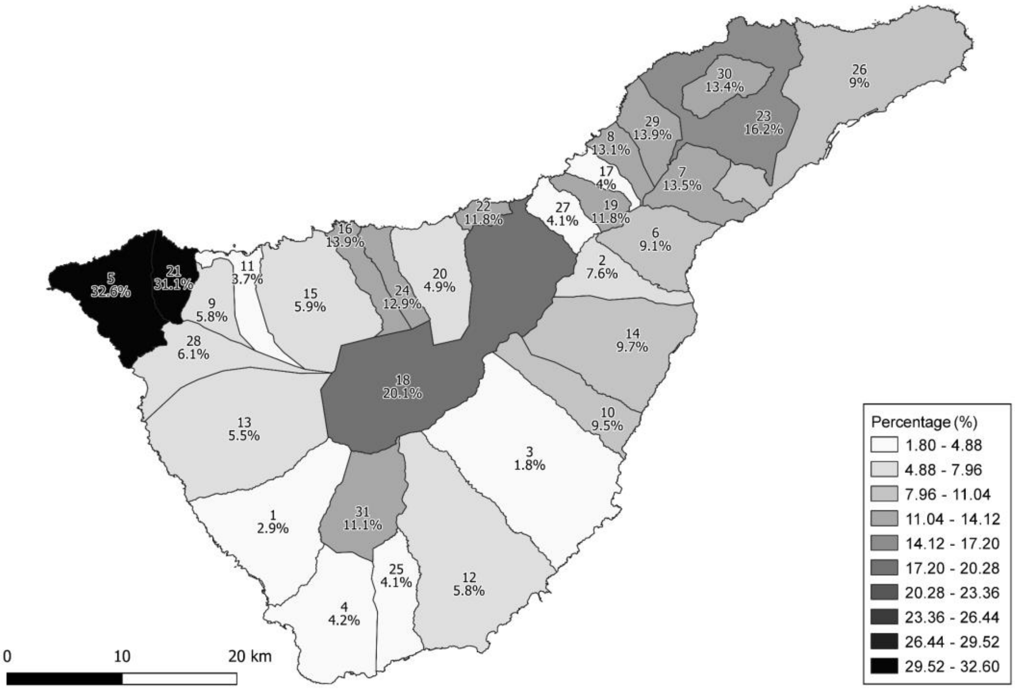

Figure S37. Population prevalence (%) of the nursing diagnosis Impaired home maintenance [NANDA-I 00098] by municipalities in the healthcare area under study.

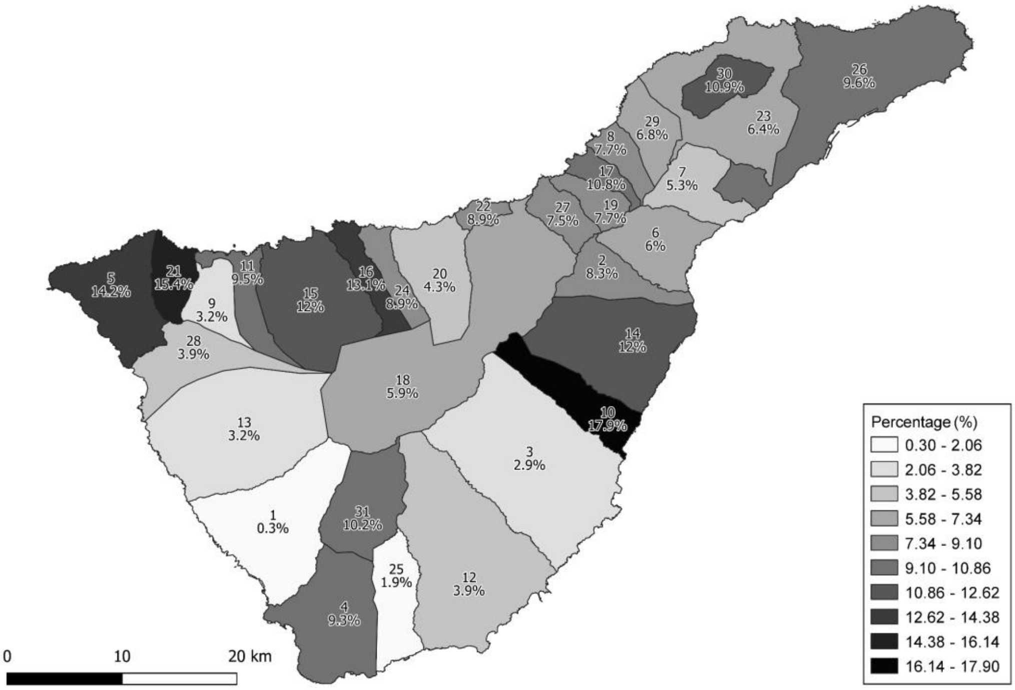

Supplement: Supplementary file 1 [file nursrep-14-00096-s001.zip › nursrep-2946181-supplementary.pdf]
